# Characterizing the microbiome of “sterile” organs in experimental mice and evidence of translocation of bacteria from the gut to other internal organs

**DOI:** 10.1002/imt2.70081

**Published:** 2025-09-22

**Authors:** Ming Xu, Shuyun Guan, Chaoran Zhong, Mingyang Ma, Li Tao, Guanghua Huang

**Affiliations:** ^1^ Shanghai Institute of Infectious Disease and Biosecurity, State Key Laboratory of Genetics and Development of Complex Phenotypes, School of Life Sciences, Department of Laboratory Medicine, Department of Infectious Diseases, Huashan Hospital Fudan University Shanghai China; ^2^ College of Pharmaceutical Sciences Southwest University Chongqing China

## Abstract

Using culturomics and metagenomics, we demonstrate the existence of non‐pathogenic microbiota in the internal organs of healthy experimental mice, challenging the traditional dogma of organ sterility. Based on the analysis of 104 commercially sourced mice (C57BL/6J, BALB/c, ICR), the study reveals that over 20% of the analyzed mice harbored a high microbial burden in the internal organs and identified a total of 463 microbial species. Several species, including *Ligilactobacillus murinus*, *Alcaligenes faecalis*, *Micrococcus luteus*, *Pseudochrobactrum asaccharolyticum*, *Escherichia coli*, and *Microbacterium* sp., were frequently identified and were abundant in the mouse tissues. Further investigation implies that microorganisms in the “sterile” tissues could be associated with the gut microbiota. Given the wide use of experimental mice in medical and biological research, these findings of resident microorganisms in the animal's internal organs raise concerns about potential variability in experimental outcomes.

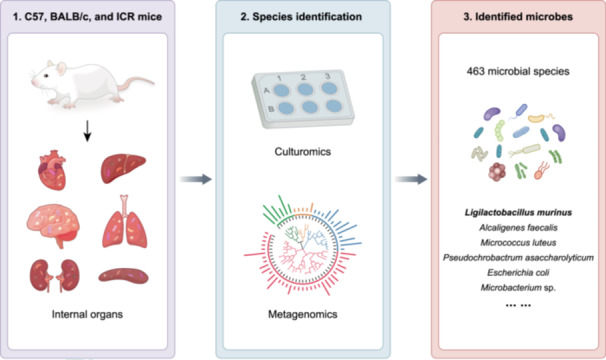

Myriads of microorganisms, collectively referred to as the microbiome or microbiota, colonize the skin, mucosal surfaces, and the digestive and genital tracts of mammals, including humans [1, 2, 3, 4]. The microbiome has significant positive and negative effects on human health, influencing processes such as nutrient absorption, immune system development and regulation, infection prevention, and disease progression [5, 6, 7]. Traditionally, internal organs and tissues were believed to be sterile [8]. However, recent studies have challenged this notion, demonstrating that the presence of living microorganisms in the organs of both healthy and diseased individuals [9, 10, 11].

Evidence suggests that intratumor microbiomes may influence the initiation, progression, metastasis, and therapeutic responses of various cancers [12, 13]. For instance, unique microbial signatures have been identified in tumor tissues of cancer patients [11]. Leinwand et al. (2022) recently reported the presence of microorganisms in the liver and their role in modulating hepatic immunity and tolerance [14]. Intratissue or intratumor microbes may originate from adjacent normal tissues (NATs) [15] or result from gut bacterial translocation, as observed in pancreatic ductal adenocarcinoma [9].

Experimental animals are widely used in biological and medical research. However, despite standardized specific‐pathogen‐free housing of inbred strains, unexplained interindividual variability persists [16]. These variations are thought to be influenced by uncontrollable environmental factors, particularly microbiota [17]. Consistently, researchers have detected living microorganisms in the liver and low levels of bacterial 16S rDNA sequences in the “sterile” organs of laboratory mice or rats [14, 18].

In this study, we used culturomics and metagenomics approaches to investigate the microbiomes of “sterile” organs along with the lung in 104 mice. We found that over 20% of the mice harbored significant microbe burdens [>1 × 10^4^ colony‐forming unit (CFU)/g tissue]. Metagenomics analysis together with germ‐free mouse model assays suggest that microbial cells could be translocated from the gut to mesenteric lymph nodes (MLNs) and then to remote tissues. These findings highlight the variability in microbiomes among laboratory mice and underscore the importance of considering these factors when interpreting experimental outcomes.

## Concept and research design for microbiome analysis of experimental mouse organs

**Figure 1:**
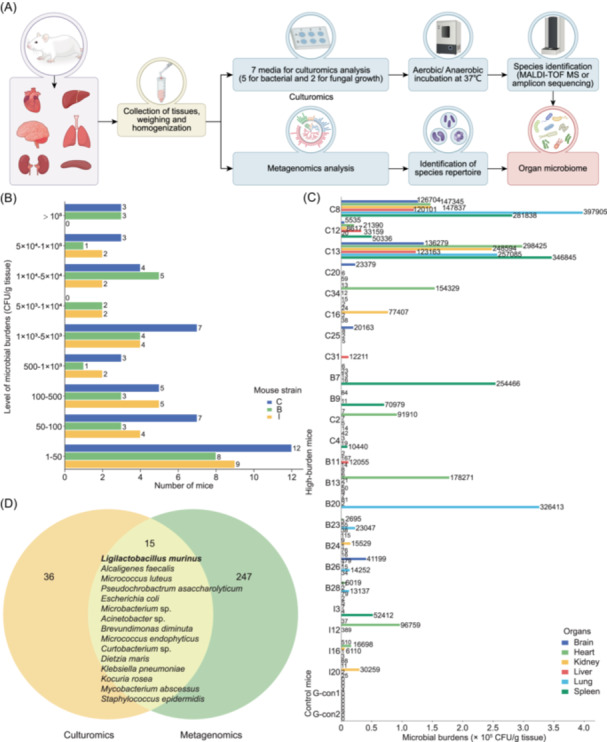
Summary of the workflow and outcomes. (A) A schematic workflow. Culturomics and metagenomics analyses of the six organs from 104 mice (C57BL/6J, BALB/c, ICR; three providers: WTL, NMO, ZYU) (Table S1). (B) Number of mice with different levels of microbial burdens based on culturomics assays. The figure shows the microbial burden levels in at least one of six organs. CFU, colony‐forming unit. CFU levels (CFU/g tissue), mouse strains, and the number of mice with varying microbial abundance are indicated. The column colors represent specific mouse strains. (C) Distribution and abundance of microbes in the organs of 23 high‐burden mice (>10^4^ CFU/g tissue) detected by culturomics. Two germ‐free mice were used as negative controls. The column colors represent specific organs, with the numbers on the columns indicating the raw CFU values, and are independent of the x‐axis scale. (D) Comparative analysis of the microbial species in the 10 high‐burden mice detected by culturomics and metagenomics assays. Strains: C, C57BL/6J; B, BALB/c; I, ICR; G‐con, Germ‐free control (C57BL/6J). This figure is associated with Figures S2, S3 and Tables S3, S4, S6.

This study aimed to determine whether microorganisms exist in the “sterile” organs of experimental mice, a widely used model in biological and biomedical research. We employed culturomics and metagenomics to identify viable microorganisms in the “sterile” organs (brain, heart, kidney, liver, spleen) and the lung tissue of mice (Figure 1A). A total of 104 mice (44 C57BL/6J, 30 BALB/c, and 30 ICR) from three Chinese providers (WTL, NMO, and ZYU) were included (Figure 1B and Table S1). All mice were healthy with no observed debilitating phenotypes. Mice were humanely euthanized, strictly disinfected with 75% ethanol, and dissected under sterilized conditions. The dissected tissues were washed carefully with 75% ethanol and rinsed with sterile phosphate‐buffered saline three times. In total, 624 organ samples were collected for microbiome analyses, including culturomics and metagenomics approaches. Parallel environmental negative controls were performed to rule out contamination originating from the laboratory. Culturomics analysis was performed on all 104 mice using seven optimal media under both aerobic and anaerobic conditions (Table S2). The dominant microbial species in different organs were analyzed in 23 mice (22.1%) with >1 × 10^4^ CFU/g tissue in at least one organ. The tissues of germ‐free mice served as the negative controls (Figure 1C). Metagenomics analysis was conducted on 10 mice with the highest microbial abundance identified by culturomics (C8, C12, C13, C16, C20, C25, C31, C34, B7, and B9). No‐tissue controls were included in DNA extraction and sequencing runs to monitor potential contamination. Contamination was well controlled throughout the entire experimental process. Finally, a comparative analysis of the microbial species detected by culturomics and metagenomics was conducted (Figure 1D).

## Culturomics assays identify viable microorganisms in the mouse organs

The microbial burden (CFU) in the brain, heart, kidney, liver, lung, and spleen tissues of each mouse was assessed by quantifying colonies on seven optimal media (Figures 1C, and S1, S2, S3, and Table S3). Among the 104 mice tested, 6 (5.8%) exhibited microbial loads exceeding 1 × 10^5^ CFU/g tissue, 17 (16.4%) had loads between 1 × 10^4^ and 1 × 10^5^ CFU/g tissue, and 19 (18.3%) had loads ranging from 1 × 10^3^ to 1 × 10^4^ CFU/g tissue in at least one organ (Figures 1B and S2, S3). Overall, 23 mice (22.1%) showed high microbial burden (>1 × 10^4^ CFU/g tissue) in at least one organ, while 42 mice (40.4%) exceeded 1 × 10^3^ CFU/g tissue in at least one organ. Additionally, eight mice exhibited CFU values exceeding 1 × 10^3^ CFU/g tissue in at least two organs (Figures S2 and S3). For the C57BL/6J mice, 19 mice had relatively low microbial burden, with CFU values below 1 × 10^2^ CFU/g tissue across all six organs, while 8 had CFU values ranging from 1 × 10^2^ to 1 × 10^3^ CFU/g tissue in at least one organ. In contrast, 11 mice (45.8%) from the WTL provider, 5 (50.0%) from the NMO provider, and 1 (10.0%) from the ZYU provider exhibited CFU values exceeding 1 × 10^3^ CFU/g tissue in at least one organ. Two mice (C8 and C13) showed particularly high CFU values (>1 × 10^5^ CFU/g tissue) across six organs, while one mouse (C12) had similar high CFU values (>1 × 10^5^ CFU/g tissue) in three organs. Additionally, two mice (C2 and C34) exhibited high CFU values (~1 × 10^5^ CFU/g tissue) in the heart, and two others (C20 and C25) had elevated CFU (>1 × 10^4^ CFU/g tissue) in the brain. Comparable microbial burdens were detected in the organs of two other mouse strains, BALB/c and ICR (Figure S3 and Table S3). Collectively, these findings demonstrate the presence of viable microbes in the organs traditionally considered “sterile” in experimental mice. However, microbial abundances varied across organs and individual mice depending on the commercial providers, mouse strains, or batches.

## Distribution and abundance of microbial species in mouse organs revealed by culturomics analysis

From the culture plates representing culturomics results of the “sterile” organs of 104 mice, a total of 2600 colonies were selected for species identification. Two representative colonies from each colony phenotype grown on individual medium plates were analyzed using matrix‐assisted laser desorption/ionization time‐of‐flight mass spectrometry (MALDI‐TOF MS) assays. For colonies that could not be accurately identified due to technical limitations, molecular identification through amplicon sequencing was performed. In total, 216 microbial species were identified, including 213 bacterial and 3 fungal species (Table S3). The composition of microbial species varied significantly among individual mice. Three mice (C3, C31, B6) harbored only a single microbial species across their six organs, while 25 mice carried between 2 and 5 species, and the remaining mice hosted more than 5 species. Notably, mice B11, B26, and I12 exhibited the highest diversity, harboring 28, 24, and 21 microbial species, respectively. Of the total 216 microbial species, 56 (25.9%) were detected in at least three mice of the 42 mice with a relatively high microbial burden (>1 × 10^3^ in at least one organ) (Figure S4). For example, *Staphylococcus epidermidis* was detected in 23 mice (54.8%), *Micrococcus luteus* in 20 mice (47.6%), *Ligilactobacillus murinus* in 16 mice (38.1%), *Staphylococcus hominis* and *Bacillus cereus* in 15 mice (35.7%), *Micrococcus endophyticus* in 11 mice (26.2%), *Microbacterium* sp. in 10 mice (23.8%), *Lysinibacillus* sp. in 9 mice (21.4%), *Lactobacillus johnsonii* in 8 mice (19.0%), and *Alcaligenes faecalis* in 7 mice (16.7%) (Figure S4, Table S4). Among the 56 bacterial species, 45 (80.4%) were Gram‐positive, including 26 Bacillota, 19 Actinomycetota. The remaining 11 (19.6%) Gram‐negative species belong to the phylum Pseudomonadota (Table S4). High‐abundance species were distributed across three phyla: Pseudomonadota, Actinomycetota, and Bacillota (Figure S5). Furthermore, a spectrum of organ‐preferential and organ‐enriched species was identified (Figures S6, S7 and Table S5).

## Metagenomics analysis of the organ microbiomes of experimental mice

Since many microbial species are unculturable in vitro and culture‐based methods poorly estimate microbial abundance, we performed metagenomics analysis using a streamlined restriction site‐associated DNA sequencing for Microbiome (2bRAD‐M) strategy to profile the microbiomes of mouse organs. We focused on the brain, heart, kidney, liver, lung, and spleen tissues from 10 mice with relatively high microbial abundance as revealed by culturomics assays. A total of 262 microbial species were identified across these six organs (Table S6). Multiple overlapping species were detected by both metagenomics and culturomics, including *Acinetobacter* sp., *Alcaligenes faecalis*, *Escherichia coli*, *L. murinus*, *Microbacterium* sp., *Micrococcus luteus*, and *Pseudochrobactrum asaccharolyticum*. Among these species, *L. murinus* exhibited a high relative abundance (Table S6). Metagenomics analysis also revealed microbial diversity across the 10 mice. For example, mice C34 and C20 harbored 123 and 76 microbial species, respectively, while mice C25 and C31 had only 11 and 17 microbial species, respectively (Table S6). In total, 97 microbial species (37.0%) were detected with a relative abundance >10^−4^ in at least two mice. Those species were distributed across 7 phyla, 11 classes, 25 orders, 39 families, 74 genera, and 97 species. The identified phyla included Bacillota (30/97, 30.9%), Pseudomonadota (21/97, 21.7%), Bacteroidota (24/97, 24.7%), Actinomycetota (19/97, 19.6%), Ascomycota (1/97, 1.0%), Deinococcota (1/97, 1.0%), and Desulfobacterota (1/97, 1.0%). Despite the distinct microbial diversity observed in each mouse, several species were common across the three mice (C8, C12, and C13). For example, *Alistipes* sp., *Anaerotruncus* sp., *Duncaniella dubosii*, *Duncaniella muris*, *Eubacterium* sp., *L. murinus*, *Muribaculum arabinoxylanisolvens*, *Muribaculum intestinale*, *Paramuribaculum intestinale*, and *Rothia mucilaginosa* were detected. Of these, *Alistipes* sp. and *Duncaniella dubosii* were detected in more than 8 mice (Figure S8, Table S6). Additionally, microbial species distribution exhibited organ‐specific patterns (Figure S9, Table S7). Metagenomics assays identified a much broader range of microbial species compared to culturomics assays, likely due to the fact that some species are difficult to culture. Furthermore, metagenomics can detect both living and dead microbes, whereas culturomics only identifies living, culturable species.

## Evidence of translocation of bacterium *L. murinus* cells from the gut to the “sterile” organs in a germ‐free mouse model

**Figure 2:**
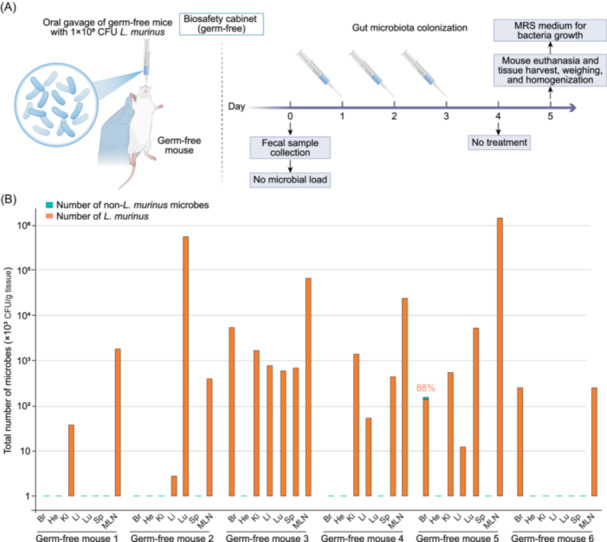
Oral gavage assays and translocation of *L. murinus* to the “sterile” organs and MLNs using a germ‐free mouse model. (A) Schematic workflow. Eight germ‐free C57BL/6J mice (7 weeks, male) were used. The absence of microbial load was confirmed through fecal collection and microbiological analysis performed on Day 0. Six germ‐free mice were orally inoculated with 1 × 10^8^ *L. murinus* cells per day for three consecutive days. Control mice were fed with sterile phosphate‐buffered saline (PBS) (*n* = 2). At 48 h post‐final gavage, mice were anesthetized. Organs (heart, lung, liver, spleen, kidney, brain, and MLNs) were harvested and used for CFU assays. de Man, Rogosa‐Sharpe (MRS) agar medium was used for *L. murinus* growth. (B) Number of *L. murinus* detected in the different gavage mouse tissues and MLNs. CFU, colony‐forming unit. Columns represent the number of microbes (CFU/g tissue) detected in the organs. Orange column, *L. murinus*; Green column, other microbes. The percentages on the columns indicate the proportion of *L. murinus* CFU (unlabeled columns represent 100% *L. murinus*). Brain (Br), heart (He), kidney (Ki), liver (Li), lung (Lu), spleen (Sp), and mesenteric lymph nodes (MLNs). Detailed information for the mice, microbial species, and microbial burden is provided in the Source Data file.

The gut of animals harbors a diverse community of microbes. To investigate whether there is a potential link between the gut microbiomes and organ microbiomes, we performed metagenomics analysis on the fecal microbiomes of mice. We focus on the 10 mice that exhibited a relatively high microbial abundance in the brain, heart, kidney, liver, lung, and spleen tissues. As shown in Figure S10, 40 microbial species were detected in both the organ and fecal microbiomes. Specifically, *L. murinus* exhibited high abundance in both organs and feces and was most frequently and abundantly detected in the MLNs (Figures S11 and S12). These findings suggest a potential translocation of microbial cells from the gut to the “sterile” organs. To further investigate this, we performed an oral gavage assay with *L. murinus* in a germ‐free mouse model (Figure 2A). Six germ‐free C57BL/6J mice were orally fed with 1 × 10^8^ CFU *L. murinus* cells daily for three consecutive days. Forty‐eight hours after the last oral gavage with *L. murinus* cells, the abundance of *L. murinus* in the different organs and MLNs was examined (Figure 2A). *L. murinus* was detected in at least two organs of all treated mice (Figure 2B). Consistent with the results in Figure S11, *L. murinus* was detected in MLNs of all the treated mice. In five out of six mice, the abundance of *L. murinus* in the MLNs was higher than that in the other “sterile” organs. *L. murinus* was the sole bacterium identified in nearly all the organs. One exception was that the brain of one mouse carried 88% of *L. murinus* and 12% of *Staphylococcus epidermidis*. As expected, a very few other microbial species, *Micrococcus* sp. (4 CFU/g liver tissue) and *Staphylococcus* sp. (9 CFU/g liver tissue), were detected in the two control germ‐free mice with low burdens, respectively (**Source data**). Although these may reflect minor contamination, they do not affect the conclusion regarding *L. murinus* dissemination. These results suggest that *L. murinus* cells could be first transferred from the gut to MLNs and then to the other “sterile” organs. This is consistent with the previous findings that translocation of microorganisms from the gut microbiota to MLNs and then to the other organs occurs prevalently in both humans and mice [19, 20].

In summary, our findings reveal the widespread presence of living microbiomes in the traditionally considered “sterile” organs of laboratory mice with different genetic backgrounds. The use of both culturomics and metagenomics assays suggests a potential link between the organ and gastrointestinal (GI) microbiomes. The presence and variation of microbes in different mouse individuals may exert physiological effects and confound experimental outcomes, raising a caveat about the interpretation of results.

## AUTHOR CONTRIBUTIONS

**Ming Xu**: Methodology; software; writing—original draft; formal analysis; validation; conceptualization; resources. **Shuyun Guan**: Methodology; validation; formal analysis; resources. **Chaoran Zhong**: Methodology; validation; formal analysis; resources. **Mingyang Ma**: Methodology; validation; formal analysis. **Li Tao**: Conceptualization; software; formal analysis; validation; writing—original draft; writing—review and editing; resources; project administration; supervision; funding acquisition; methodology. **Guanghua Huang**: Conceptualization; software; validation; formal analysis; supervision; funding acquisition; project administration; resources; writing—original draft; writing—review and editing. All authors have read the final manuscript and approved it for publication.

## CONFLICT OF INTEREST STATEMENT

The authors declare no conflicts of interest.

## ETHICS STATEMENT

All animal experiments were conducted in compliance with the guidelines of the Animal Care and Use Committee of Fudan University (2021JS004). Ethical approval for this study was obtained from the same committee.

## Supporting information


**Figure S1.** Representative examples of culture plates and FISH images for microbial detection in mouse brain, heart, kidney, liver, lung, and spleen tissues.
**Figure S2.** Abundance of microbes detected in the brain, heart, kidney, liver, lung, and spleen tissues of C57BL/6J mice based on culturomics assays.
**Figure S3.** Abundance of microbes detected in the brain, heart, kidney, liver, lung, and spleen tissues of BALB/c, ICR and germ‐free mice based on culturomics assays.
**Figure S4.** Composition and abundance of microbial species isolated by culturomics assays from six organ tissues of mice with a high microbial burden.
**Figure S5.** Phylogenetic tree of the representative species enriched in the mouse brain, heart, kidney, liver, lung, and spleen tissues.
**Figure S6.** Unique and overlap species among different organs of 42 mice with a microbial burden higher than 10^3^ CFU/g tissue.
**Figure S7.** Most frequently isolated microbial species from different organs of the 23 mice with a high microbial burden.
**Figure S8.** Composition and abundance of microbial species in the 10 mice with high microbial abundance detected by metagenomics assays.
**Figure S9.** Taxonomic composition of microbes in each organ of the 10 high‐burden mice detected by metagenomics assays.
**Figure S10.** Comparative analysis of the microbiomes between the organs and feces detected in 10 high‐burden mice.
**Figure S11.** The distribution of *L. murinus* in the mouse organs and MLNs.
**Figure S12.** Representative examples of culture phenotype for *Ligilactobacillus murinus* detection in the organs of the naturally microbe‐burden mice.

**Table S1.** Detailed information of the mice used in this study.
**Table S2.** Summary of the culture media used in this study.
**Table S3.** Information of the microbial species isolated from the mouse organs by culturomics.
**Table S4.** The microbial species detected in the organs of at least three mice among 42 mice by culturomics.
**Table S5.** The dominate species isolated from different organs by culturomics.
**Table S6.** The microbial species detected by metagenomics assays.
**Table S7.** Taxonomic composition of microbes in each organ of the 10 mice detected by metagenomics assays.

## Data Availability

The authors declare that the data supporting the findings of this study are included in the article and its Supporting Information files. All raw sequencing data generated in this study have been deposited in the NCBI Sequence Read Archive under accession number PRJNA1193792 (https://www.ncbi.nlm.nih.gov/bioproject/?term=PRJNA1193792). Source data for Figures 2, S2, S3, S4, S8, S9, and S11 are provided in the Supporting Information. Supplementary materials (methods, figures, tables, additional results and discussion, graphical abstract) may be found in the online DOI or iMeta Science http://www.imeta.science/.
