# Supplementary material for: Characterizing the microbiome of “sterile” organs in experimental mice and evidence of translocation of bacteria from the gut to other internal organs
Source: Imeta. 2025 Sep 22;4(5):e70081. doi: 10.1002/imt2.70081 (PMC12527995; doi:10.1002/imt2.70081)
Supplement: Supplementary file 1 — Figure S1. Representative examples of culture plates and FISH images for microbial detection in mouse brain, heart, kidney, liver, lung, and spleen tissues. Figure S2. Abundance of microbes detected in the brain, heart, kidney, liver, lung, and spleen tissues of C57BL/6J mice based on culturomics assays. Figure S3. Abundance of microbes detected in the brain, heart, kidney, liver, lung, and spleen tissues of BALB/c, ICR and germ‐free mice based on culturomics assays. Figure S4. Composition and abundance of microbial species isolated by culturomics assays from six organ tissues of mice with a high microbial burden. Figure S5. Phylogenetic tree of the representative species enriched in the mouse brain, heart, kidney, liver, lung, and spleen tissues. Figure S6. Unique and overlap species among different organs of 42 mice with a microbial burden higher than 103 CFU/g tissue. Figure S7. Most frequently isolated microbial species from different organs of the 23 mice with a high microbial burden. Figure S8. Composition and abundance of microbial species in the 10 mice with high microbial abundance detected by metagenomics assays. Figure S9. Taxonomic composition of microbes in each organ of the 10 high‐burden mice detected by metagenomics assays. Figure S10. Comparative analysis of the microbiomes between the organs and feces detected in 10 high‐burden mice. Figure S11. The distribution of L. murinus in the mouse organs and MLNs. Figure S12. Representative examples of culture phenotype for Ligilactobacillus murinus detection in the organs of the naturally microbe‐burden mice. [file IMT2-4-e70081-s002.docx]

**Supporting Information** **to**

# Characterizing the Microbiome of 'Sterile' Organs in Experimental Mice and Evidence of Translocation of Bacteria from the Gut to Other Internal Organs

**Running title**: Microbiome of mouse “sterile” organs

Ming Xu^1#^, Shuyun Guan^1#^, Chaoran Zhong^1#^, Mingyang Ma^1^, Li Tao^1^*, and Guanghua Huang^1,2^*

^1^ Shanghai Institute of Infectious Disease and Biosecurity, State Key Laboratory of Genetics and Development of Complex Phenotypes, School of Life Sciences, Department of Laboratory Medicine, Department of Infectious Diseases, Huashan Hospital, Fudan University, Shanghai 200438, China

^2^ College of Pharmaceutical Sciences, Southwest University, Chongqing 400716, China

^#^ These authors contributed equally: Ming Xu, Shuyun Guan, Chaoran Zhong

***Correspondence:**

**Guanghua Huang** (ORCID: 0000-0002-4761-7548)

State Key Laboratory of Genetic Engineering, School of Life Sciences, Fudan University, Shanghai 200438, China. E-mail: [huanggh@fudan.edu.cn](mailto:huanggh@fudan.edu.cn)

**Li Tao** (ORCID: 0000-0001-6109-3480)

State Key Laboratory of Genetic Engineering, School of Life Sciences, Fudan University, Shanghai 200438, China. E-mail: [taoli@fudan.edu.cn](mailto:taoli@fudan.edu.cn)

## Additional Results

### Experimental evidence of viable microorganisms in the mouse organs

As shown in **Figure S1A**, distinct microbial colonies formed on the plates when ground tissues were plated onto media and incubated under either aerobic or anaerobic conditions. To confirm the presence of microbes in the mouse organs, we performed fluorescence *in situ* 16S rRNA-hybridization (FISH) assays, which revealed clear fluorescence signals in all tested tissues (**Figure S1B**). Notably, despite high microbial loads in some organs, colony-forming unit (CFU) values in mouse blood were consistently very low (**Table S3**). To determine whether the organ microbiome changes with age, we analyzed the organ microbial burden in mice aged 4, 6, 7 weeks, or 3, 12, 21 months, respectively (**Table S1**). Nevertheless, no clear association was observed between organ microbial abundance and the age of the mice (**Figure S2 and Table S3**). Further studies are needed to address these questions.

Importantly, similar microbial burdens were observed in the organs of two other mouse strains, BALB/c and ICR, obtained from the WTL, NMO, and ZYU commercial experimental animal providers (**Figure S3 and Table S3**). Notably, the microbial loads in C57 and ICR mice purchased from ZYU were lower compared to those from WTL and NMO. These results demonstrate that the presence of viable microbes in traditionally “sterile” organs is prevalent across diverse mouse strains. Nevertheless, microbial abundance varied by commercial providers or batches.

### Microbial species in mouse organs revealed by culturomics analysis

Phylogenetic analysis based on 16S rDNA sequences revealed that high-abundance species identified by culturomics were distributed across three phyla: Pseudomonadota, Actinomycetota, and Bacillota (**Figure S5 and Table S4)***.* For example, the Pseudomonadota phylum included *Alcaligenes faecalis*, *Brevundimonas diminuta*, and *Acinetobacter* sp., while the Actinomycetota phylum contained *Microbacterium* sp. and *Micrococcus* sp. The Bacillota phylum included high-abundance species such as *Staphylococcus* sp., *Bacillus* sp., and *Ligilactobacillus murinus*.

The distribution of dominant microbial species also varied among different mouse strains. The most frequently isolated microbial species from the organs of C57BL/6J, BALB/c, ICR mice are shown in **Figure S4, Tables S3** and **S4**. For example, *Alcaligenes faecalis*, *Brevundimonas diminuta*, and *Pseudochrobactrum kiredjianiae* were dominant in C57BL/6J mice, while *Lysinibacillus fusiformis* and *Corynebacterium tuberculostearicum* were found in both C57BL/6J and BALB/c mice. In contrast, *Micrococcus luteus* was dominant in C57BL/6J and ICR mice, and *Staphylococcus epidermidis* was common in BALB/c and ICR mice (**Figure S4**).

Further analysis was performed to determine microbial distribution and abundance in the six organs of different mice. An UpSet plot analysis was performed to identify species that were unique or shared among different organs based on the analysis of the 42 mice with a relatively high microbial burden (> 1 × 10^3^ CFU/g tissue in at least one organ). Microbial species isolated from the same organ of at least three individual mice were analyzed. As shown in **Figure S6**, 9 microbial species were found to be unique in a particular organ, whereas 13 species were shared across different organs. Three species, *Paracoccus yeei*, *Acinetobacter lwoffii*, and *Moraxella osloensis*, were only found in the liver, whereas *Dietzia maris*, *Limosilactobacillus reuteri*, and *Pseudochrobactrum kiredjianiae* were only found in the kidney. *Acinetobacter* sp., and *Escherichia coli* were unique in the lung, and *Curtobacterium* sp. was unique in the heart. *L. murinus*, *Staphylococcus hominis*, *Bacillus cereus*, and *Micrococcus luteus* were identified in four organs, while *Mammaliicoccus lentus*, *Lysinibacillus* sp., and *Lactobacillus johnsonii* were found in three organs. Furthermore, we analyzed the dominant species among different organs in the 23 mice with a high microbial burden (> 10^4^ CFU/g tissue in at least one organ). As shown in **Figure S7,** *Alcaligenes faecalis* and *Brevundimonas diminuta* were dominant in all organs except the lung, while *Staphylococcus* sp. was dominant in all organs except the heart. *Alcaligenes faecalis* was the most dominant species in the brain, spleen, kidney, and liver. Additionally, *Micrococcus luteus* were dominant in the brain, heart, and spleen, while *Bacillus* sp. was enriched in kidney, liver, and lung (**Figure S7, Table S5**). Taken together, the six organs exhibited unique microbial signatures, and individual mice displayed similar distribution pattern. The dominant species identified in the 23 high-burden mice were also prevalent in a broader population. For example, *Micrococcus luteus* was found in 47.6% of the mice (20/42), *L. murinus* in 38.1% (16/42), *Mammaliicoccus lentus* in 21.4% (9/42), and *Alcaligenes faecalis* in 16.7% (7/42).

### Metagenomics analysis of the organ microbiomes of experimental mice

We further investigated whether the microbial species distribution exhibited organ specificity based on the metagenomics data. As shown in **Figure S9A and Table S7**, distinct microbiome features were observed across different mouse organs, with specific phyla predominating in each organ. For example, Bacteroidota, Pseudomonadota, and Bacillota were predominant in the liver, while Bacillota was the most abundant phylum in the lung. The kidney and brain microbiomes were enriched in Bacteroidota, and Bacillota, while the heart microbiome exhibited a higher burden of Acidobacterioa, Actinomycetota, and Bacteroidota.

At the genus level, species distributions also exhibited organ-specific patterns (**Figure S9B and Table S7**). For example, *Alcaligenes*, *Rothia*, *Brevundimonas*, and *Pseudochrobactrum* were enriched in the brain, while *Duncaniella, Acinetobacter*, and *Mycobacterium* were predominant in the heart. In the kidney, *Rothia*, *Ligilactobacillus*, and *Alistipes* were common, while *Ligilactobacillus*, *Alistipes*, *Sphingomonas*, and *Bordetella* were enriched in the liver. In the lung, *Alistipes*, *Streptococcus,* and *Burkholderia* were dominant, and in the spleen, *Pseudomonas*, *Corynebacterium*, and *Alcaligenes* were the most prevalent genera. Consistently, *Ligilactobacillus* species were also enriched in the kidney and liver, while *Alcaligenes* and *Acinetobacter* species were identified in the brain and heart by culturomics assays.

At the species level, distinct dominant species were identified in different organs (**Table S7**). For example, *Muribaculum arabinoxylanisolvens, Alistipes* sp., and *Duncaniella dubosii,* were enriched in all six organs, while *L. murinus*, *Duncaniella muris*, and *Eubacterium* sp., were abundant in the brain, kidney, liver, lung, and spleen. *Muribaculum intestinale*, *Duncaniella* sp., *Paramuribaculum intestinale*, and *Anaerotruncus* sp., were abundant in four organs, while *Kineothrix* sp., *Acetatifactor* sp., and *Parabacteroides gordonii*, were enriched in three organs. *Ralstonia pickettii,* *Escherichia coli,* *Alcaligenes faecalis,* *Faecalibaculum rodentium* was enriched in the brain, heart, liver, and spleen, respectively. *Alistipes* sp., and *Duncaniella dubosii* were detected in more than 8 mice by metagenomics assays (**Figure S8, Table S6**). In conclusion, metagenomics analysis has revealed the presence of microbial species in the "sterile" organs of experimental mice that are difficult to culture. These findings suggest that species such as *Alistipes sp.*, *Duncaniella dubosii*, and others may be dominant in these organs but are not readily identified through traditional culturomics approaches.

### A potential link between the fecal and organ microbiomes

To investigate a potential link between the gut microbiome and the microbiome of “sterile” organs, we performed metagenomics analysis on the fecal microbiomes of mice. As shown in **Figure S10A**, four common phyla (15.4%) were abundant in both fecal and organ microbiomes. These included Bacteroidota, Bacillota, Actinomycetota, and Pseudomonadota. Notably, Pseudomonadota and Actinomycetota species were more prevalent in the organ microbiomes, while Bacillota and Bacteroidota species were enriched in the fecal microbiomes. Additionally, Basidiomycota was identified as a dominant phylum common to the microbiomes of the six organs, but not the fecal microbiomes.

At the genus level, we analyzed the top 30 abundant taxa in the fecal microbiomes, as shown in **Figure S10B**. Of these, 10 genera (33.3%) were also detected in the microbiomes of the six organs. These included *Muribaculum*, *Duncaniella*, *Alistipes*, *Ralstonia*, *Rothia*, *Choladocola*, *Acetatifactor*, *Paramuribaculum*, *Pelethomonas*, and *Faecalibaculum*. Among these, *Muribaculum* and *Ralstomia* were more abundant in the organ microbiomes, while *Alistipes*, *Faecalibaculum*, *Pelethomonas*, and *Paramuribaculum* were more abundant in the fecal microbiomes. Notably, *Cutibacterium* species were specifically enriched in the organ microbiomes (**Figure S10B**). We then performed an UpSet plot analysis to explore the overlap of microbial species between the organ and fecal microbiomes (**Figure S10C**). The fecal microbiomes showed the highest diversity, while the liver tissue microbiome had the lowest diversity. Tissue-specific microbial species were also identified in the six organs (**Figure S10C**). Furthermore, nine species, including *Alistipes* sp., *Anaerotruncus* sp., *Duncaniella dubosii*, *Duncaniella muris*, *Eubacterium* sp., *L. murinus*, *Muribaculum arabinoxylanisolvens*, *Muribaculum intestinale*, and *Paramuribaculum intestinale*, were dominant in both the organs and feces. Alpha diversity measures indicated a significantly lower richness of species in the organ microbiomes compared to the feces (**Figure S10D**). Weighted principal coordinate analysis (PCoA) based on species-level β-diversity confirmed distinct differences in microbial composition between the organ and fecal microbiomes (**Figure S10E**). These results suggest that, compared to the fecal microbiomes, the microbial profiles of the “sterile” organs exhibit more similarity. Both common and different microbial species were identified in the organ and fecal microbiomes, suggesting that the organs may represent unique ecological niches. Moreover, since most of the intratissue microbial species could also be found in the fecal microbiota, indicating a potential link to the gut microbiota.

### Detection of *Ligilactobacillus murinus* in mouse organs and MLNs

Since *L. murinus* was one of the most frequently isolated bacterium from the mouse “sterile” organs (**Figures S11 and S12**), we next re-evaluated the abundance of microbial organisms in these tissues and mesenteric lymph nodes (MLNs) using de Man, Rogosa-Sharpe (MRS) medium (an optimal medium for the growth of *L. murinus*)*.* Ten C57BL/6J mice purchased from ZYU were analyzed as described earlier. As shown in **Figure S11**, *L. murinus* was detected in MLNs of all the 10 mice, in the brain and lung tissues of two mice, in the kidney and spleen of four mice, and in the liver of five mice. In eight out of ten mice, the abundance of *L. murinus* in the MLNs was higher than that in the other organs. In the heart tissue, the bacterial burdens were extremely low and no *L. murinus* cells were detected. These findings suggest translocation of *L. murinus* from the gut microbiota to MLNs, with subsequent dissemination to historically “sterile” organs.

## Additional Discussion

Experimental mice are widely used as animal models in biological and biomedical research due to their high physiological and genetic similarity to humans, small size, rapid reproductive cycle, and ease of maintenance in laboratory environments [1, 2]. Although most commonly used strains are inbred mice with very limited genetic variation, environmental and non-genetic factors, such as commensal microbes, can still contribute to phenotypic or experimental variability among individual mice. In this study, we aimed to explore whether living microorganisms are present in traditionally considered “sterile” organs of laboratory mice, and if so, how the organ microbiomes could influence the physiological and phenotypic variability of individual mice and the outcomes of research studies.

We analyzed the microbiomes of six organs (brain, heart, kidney, liver, lung, and spleen) from 104 laboratory mice using both culturomics and metagenomics assays. These mice were sourced from three major experimental animal providers in China, including different batches of C57BL/6J, BALB/c, ICR strains (**Figures 1, S2, S3 and Table S1**). We found that approximately 22.1% of mice (23/104) harbored a microbial burden of more than 1 × 10^4^ CFU/g tissue in at least one organ, and 5.8% of mice (6/104) had a microbial burden greater than 1 × 10^5^ CFU/g tissue in at least one organ. Some mice exhibited high microbial levels in multiple organs (**Figures** **S2 and S3**). Notably, the inter-individual variability in microbial burden likely correlates with host immune status, a relationship requiring mechanistic exploration. Longer-term colonization studies are necessary to clarify whether microbes colonize stably or are transient, and to support conclusions regarding microbial persistence and host impact. Additionally, although high microbial loads were found in organs across a substantial male mouse, further experiments must be designed to determine the relationship between sex and microbial loads. Through culturomics, we identified 216 microbial species, while metagenomics analysis revealed 262 species. Despite variability in microbial diversity across organs, we identified a core group of microbial species present in multiple organs. Notably, several species, *L*. *murinus*, *Alcaligenes faecalis*, *Micrococcus luteus*, *Pseudochrobactrum asaccharolyticum*, *Escherichia coli*, and *Microbacterium* sp., were the most frequently identified across both assays. Nine species, including *L. murinus*, *Alistipes*_sp., *Anaerotruncus*_sp., *Duncaniella_dubosii*, *Duncaniella_muris*, *Eubacterium* sp., *Muribaculum arabinoxylanisolvens*, *Muribaculum_intestinale*, and *Paramuribaculum_intestinale*, were found to be abundant in both the organ and fecal microbiomes. Notably, the translocation of *L. murinus* cells from the gut to MLNs and the “sterile” tissues was verified by a germ-free mouse model. These findings suggest a potential link between the mouse organ and the gut, indicating possible translocation of gastrointestinal microbes to the traditionally "sterile" organs.

Environmental contamination is always of particular concern in the study of organ microbiome. Here, we have applied multiple approaches with stringent controls to avoid contaminations and to verify the presence of microbiome in the “sterile” organs, including microbial culture, metagenomic DNA sequencing, and FISH staining assays. Although our findings align with prior reports of resident microbiomes within organ parenchymal cells [3], this study is limited by the absence of definitive evidence for *in situ* microbial colonization. Specifically, direct ultrastructural visualization of intact microorganisms (e.g., via transmission electron microscopy) and functional validation through immune response assays are lacking. Consequently, the localization and functional relevance of the detected microorganisms require further investigation. To avoid potential contamination, strict disinfection assays were performed. The mice skin was comprehensively disinfected with 75% ethanol and sterile operations were performed according to aseptic-surgery protocols. The surface of the dissected tissues was washed carefully with 75% ethanol and then rinsed with sterile phosphate-buffered saline (PBS) three times. Notably, no microbes were detected in some organs of regular mice and also very few microbes (< 10 CFU/g tissue) were found in the organs of germ-free mice, suggesting that disinfection assays were performed well and environmental contamination did not occur during dissection or culture procedures (**Figure 1C**). Moreover, several obligate anaerobic species were detected by using metagenomics assay, including the *Lachnoanaerobaculum* sp., *Peptostreptococcus anaerobius*, and *Prevotella* species. These findings further indicate the real existence of microbial species in the mouse tissues since the anaerobic species could not exist in the laboratory environment or mouse skin.

Given the widespread distribution of living microbes in the organs, it raises the question: how might these microbes affect the physiology of experimental mice? Could the organ microbiomes impact the outcomes of experiments and the reproducibility of research? Increasing evidence suggests that the presence of microbiomes in various tissue may regulate the development, progression, and treatment of diseases [4−9]. For example, commensal bacteria such as *Streptococcus*, *Prevotella*, and *Veillonella* have been linked to lung function and respiratory health [10]. Dysbiosis in the lung microbiome has been associated with increased inflammation and exacerbation of respiratory symptoms [11−13]. Several studies have also reported the presence of microbiomes in tumors of the lung [4, 9], pancreas [5], liver [6], and breast tissues [7]. The microbial composition in tumors was found to be similar to that in normal adjacent tissues (NATs), suggesting that microbes in these tissues may proliferate rapidly within the unique tumor microenvironments (TMEs) [14]. However, it remains unclear how these tissues- or tumor-associated microbes influence disease progression and treatment outcomes. In our study, we demonstrated that the organs of laboratory mouse harbor a significant number of living microbes, which could serve as an invaluable model for studying the role of intratissue microbes in disease development and the interpretation of experimental results. The detection of wide distribution of *L. murinus* in the mouse organs and MLNs indicate a possible translocation of microbial cells from the gut to other “sterile” organs. This is consistent with previous studies suggesting that gut vascular barrier impairment can lead to the dissemination of intestinal bacteria and the onset of condition like cancer [15].

The C57BL/6J, BALB/c, and ICR mice used in this study are inbred animals, which are not true wild types and carry several genetic defects [16, 17]. Over a century of long-term laboratory passages may have introduced unknown genetic alterations, such as genetic shift and drift [18−20]. It remains unclear whether the intratissue microbes observed in these mice have become an integral part of their organs through long-term interactions and co-evolution between the host and its microbial inhabitants. Additionally, the immune systems of inbred mice may be more tolerant to microbial colonization in tissues. Interestingly, we did not observe significant differences in immune responses or cytokine expression, such as IL-1β, IL-6, IL-17, tumor necrosis factor α (TNF-α), and interferon-γ (IFN-γ), between mice with or without a high burden of microbes (data not shown). This suggests that the intratissue microbiomes under basal conditions did not elicit a detectable immune response. Despite that, it remains possible that the intratissue microbiomes influence the host’s immune capacity in response to subsequent challenges, such as during infection or disease initiation and progression. Pathogenic bacteria were rarely detected in the mice studied. For example, *Staphylococcus aureus* cells were identified in low abundance in five mice (C8, C12, C20, C24 and B10), while *Klebsiella pneumoniae* was detected in a single mouse (C13). Given that *S. aureus* in a common commensal in both humans and animals, its presence in small number in mouse organs is unsurprising. These findings support the hypothesis that the majority of microbes identified in mouse organs may not be pathogens but rather harmless or potentially beneficial commensals.

## Materials and Methods

### Animals

A total of 104 healthy mice (44 C57BL/6J, 30 BALB/c, 30 ICR) were purchased from three major experimental animal providers in China: WTL, NMO, and ZYU (**Table S1**). The selection of these three suppliers was based on three criteria, including regulatory compliance, quality assurance, and commercial viability. Germ-free C57BL/6J mice were provided by Shanghai Tenth People’s Hospital and bred in a gnotobiotic facility. All animal experiments were conducted in compliance with the guidelines of the Animal Care and Use Committee of Fudan University (2021JS004). Ethical approval for this study was obtained from the same committee.

### Sample collection and processing

Upon acquisition, the mice were humanely euthanized. Brain, heart, liver, spleen, lung and kidney tissues were collected for microbiome analysis. The mice were first weighed and then intraperitoneally injected with 1% pentobarbital (100 mg/kg) for anesthesia. To avoid potential contamination, strict disinfection assays were performed. The mice skin was comprehensively disinfected with 75% ethanol and sterile operations were performed according to aseptic-surgery protocols. The mice were dissected, and blood samples were carefully collected from the heart ventricles. To prevent contamination, all procedures were conducted under sterile conditions in accordance with aseptic surgery protocols. The six organs (heart, lung, liver, spleen, kidney, and brain) were harvested sequentially. The surface of the dissected tissues was washed carefully with 75% ethanol and then rinsed with sterile phosphate-buffered saline (PBS) three times. Subsequently, each organ was weighed and divided into six equal portions. One portion was immediately frozen in liquid nitrogen and stored at -80^o^C for metagenomic sequencing analysis. Another portion was fixed in 10% formalin (G2162, Solarbio, China) and stored at 4^o^C for tissue sectioning. The remaining portions were freshly ground and processed in sterile tubes with distinct liquid media for culturomics. To minimize cross-contamination, a separate set of surgical instruments was used for each organ.

Fecal samples from each mouse were collected in sterile tubes, rapidly frozen in liquid nitrogen, and stored at -80^o^C until further use.

### Culturomics

Culturomics assays were conducted following a previously described protocol with slight modifications [21]. Briefly, three tissue samples from each organ were suspended in different liquid media: Brain Heart Infusion (BHI) (aerobic condition), BHI + 0.5 g/L L-Cysteine (anaerobic condition), and Yeast Extract Peptone Dextrose (YPD) (fungal-selective condition). Negative control tubes (without tissues) were prepared for each condition to rule out potential contamination. All samples were homogenized and plated onto various agar media and incubated under aerobically or anaerobically conditions (**Table S2**). Anaerobic cultures were handled entirely within anaerobic incubators (D500G, GENE SCIENCE, USA). Environmental negative controls were included to assess laboratory-borne contamination. Based on preliminary testing of 30 culture media for culturomics, seven optimal media (BHI1, BHI2, AIA, Chocolate agar, YCFA, YPD and CDA) were selected for this study (**Table S2**).

After 5 days of incubation at 37^o^C, colonies were selected and streaked onto fresh media for species identification. A trained colony-picking approach was employed [22], with two representative colonies of each morphology (based on colony shape, color, and size) selected from each plate. Colony-forming units (CFU) of each microbial species within an organ were estimated based on similar colony phenotype on the same plate. All selected colonies were analyzed by matrix-assisted laser desorption/ionization time-of-flight mass spectrometry (MALDI-TOF MS, AUTOF MS1000 system) using standard on-target formic acid extraction [21]. For isolates that could not be accurately identified using MALDI-TOF MS, further identification was performed using amplicon sequencing analysis.

### Amplicon amplification and sequencing

Genomic DNA from each isolate was extracted using TIANamp Bacteria DNA Kit (DP302, TianGen, Beijing, China) following the manufacturer's instructions. In brief, approximately 300 μL of glass beads (G8772, Sigma-Aldrich, USA) and 200 μL of buffer GA were added to the cell pellet and homogenized using a Mini-Beadbeater-16 (607EUR, Bio-Spec, USA). Subsequently, 20 μL of protease K solution and 220 μL of buffer GB were added in sequence, followed by incubation at 70^o^C for 10 min. After two rounds of washing, the genomic DNA was isolated. The 16S rRNA gene was amplified by PCR using universal primers: 27F (5’-AGAGTTTGATCCTGGCTCAG-3’) and 1492R (5’-GGTTACCTTGTTACGACTT-3’). The resulting PCR products were sent to Sangon Biotech (Shanghai, China) for Sanger sequencing. The sequences obtained were aligned with reference type strains using the NCBI BLAST algorithm for species identification.

### Metagenomics analysis

Genomic DNA from animal organs and feces was extracted using the QIAamp Fast DNA Stool Mini Kit (51604, Qiagen, Germany) according to the manufacturer’s protocol. Briefly, the samples were suspended in 400 μL of inhibitEX buffer, along with two Steel beads (BE6638, EASYBIO, China) and ~300 μL of glass beads (G8772, Sigma-Aldrich, USA), and homogenized using a cryogenic high-throughput tissue grinder (SCIENTZ, Ningbo, China). After centrifugation at 12, 000rpm for 5 min, 15 μL of protease K solution and 200 μL of buffer AL were added to the supernatant. The mixture was incubated at 70°C for 10 min, followed by the addition of 200 μL of anhydrous ethanol. The entire mixture was then transferred to an adsorption column. After two rounds of washing with buffers AW1 and AW2, genomic DNA was eluted. Negative control samples (no tissue) underwent DNA extraction simultaneously to account for potential contamination.

Restriction site-associated DNA sequencing for Microbiome (2bRAD-M) sequencing was performed by OE Biotech (Qingdao, China) to identify microbiomes in animal tissues and feces. The 2bRAD-M library was prepared following the protocol developed by Wang *et al*. with minor modifications [23]. Briefly, genomic DNA (1 pg-200 ng) was digested with 4 U of the enzyme BcgI (NEB), and 800 U of T4 DNA ligase (NEB) was used to ligate adaptors to both ends of the DNA fragments. The ligation products were amplified, and the PCR products were resolved using 8% polyacrylamide gel. DNA bands of approximately 100 bp were excised and treated with nuclease-free water for 6-12 h at 4^o^C. Sample-specific barcodes were introduced via PCR using platform-specific barcode-bearing primers. The 20 µL PCR reaction contained 6 µL of gel-extracted PCR product, 0.2 µM of each primer, 0.3 mM dNTP, 1×Phusion HF buffer, and 0.4 U of Phusion high-fidelity DNA polymerase (NEB). PCR products were purified using the QIAquick PCR purification kit (Qiagen, Germany) and sequenced on the Illumina Nova PE150 platform (Illumina, USA).

2bRAD microbial genome database (GTDB/Ensembl, n = 404,199 genomes) was used for bioinformatic processing. Sequenced 2bRAD tags were mapped with G-score filtering to control false-positive identifications [24]. The average read coverage of 2bRAD markers per species was calculated, representing relative abundance of each species in the sample at the given sequencing depth. Mock-derived (no tissue) data were subtracted from the sample data.

### Fluorescence *in situ* hybridization (FISH)

The dissected tissues were fixed in 10% formalin and stored at 4^o^C. Tissue sectioning and staining were performed by Seryicebio Inc. (Wuhan, China). Briefly, tissue blocks from the target area were cut into ~3 mm slices, dehydrated through a gradient of alcohols (from low to high concentrations), and embedded in paraffin wax. The embedded tissues were sectioned at 10 μm intervals, with each section having a thickness of 4 μm. The sections were then deparaffinized and rehydrated sequentially. Each tissue section was hybridized with two probes simultaneously: a Cy3-labeled universal bacterial probe, EUB338 (5'-GCTGCCTCCCGTAGGAGT-3'), which fluoresced red, and a 6-FAM-labeled nonspecific complement probe (5'-CGACGGAGGGCATCCTCA-3'), which fluoresced green [15]. The nonspecific probe served as a negative control to confirm the absence of non-specific hybridization. Following hybridization, the sections were washed to remove the hybridization solution. The nuclei were counterstained with diamidino-phenyl-indole (DAPI). Finally, the sections were mounted using antifade solution (G1401, Seryicebio, Wuhan, China). Images were captured using an Ortho-Fluorescent Microscopy (NIKON ECLIPSE CI, Nikon, Japan). Fluorescence signals were observed under the following conditions: FAM (488) emitted green fluorescence with an excitation wavelength of 465-495 nm and an emission wavelength of 515-555 nm, while CY3 emitted red fluorescence with an excitation wavelength of 510-560 nm and an emission wavelength of 590 nm.

### Phylogenetic analysis

A list of 16S rRNA gene sequences was compiled from high-abundance strains identified through Culturomics. The sequences were aligned by ClustalW, and phylogenetic inference was performed using the Neighbor-Joining method in MEGA11 software. A total of 39 phylogenetic trees of high-abundance isolates were constructed. The visualization and further processing of the evolutionary trees were conducted using the iTOL platform (<https://itol.embl.de/>).

### Bacterial burden assay for *Ligilactobacillus murinus* using MRS medium

Ten C57BL/6J mice (7 weeks, male) purchased from ZYU were used for bacterial burden analysis. The six organs (heart, lung, liver, spleen, kidney, and brain) and MLNs were harvested and weighed sequentially. As described earlier, the tissues were homogenized and were plated onto the de Man, Rogosa-Sharpe (MRS) agar medium (an optimal medium for the growth of *L. murinus*) and incubated under aerobically conditions at 37^o^C (**Table S2**) [25]. The identification of bacteria was achieved using MALDI-TOF MS and 16S rRNA gene sequencing assays.

### *L. murinus* translocation assays

Eight germ-free C57BL/6J mice (7 weeks, male) were used. Prior to experimental treatment, fecal samples from the mice were collected, plated on medium plates, and no microbial load was observed. *L. murinus* was grown in MRS broth for 24 h at 37^o^C, and then collected by centrifuge and washed with sterile PBS. Bacterial cells were resuspended in sterile PBS at a concentration of approximate 5 × 10^8^ CFU/mL. 1 × 10^8^ CFU in 200 μL of PBS were used for oral gavage assays (one feeding per day for 3 consecutive days). Control mice were orally gavaged with sterile PBS. At 48 h post-final gavage, mice were anesthetized. Organs (heart, lung, liver, spleen, kidney, brain, and MLNs) were harvested and weighed sequentially. After washing once with 75% ethanol and rinsing with sterile PBS three times, the organs were homogenized, and plated onto MRS agar medium, and incubated under aerobically conditions at 37^o^C (**Table S2**) [25]. All procedures were performed in a cleaned biosafety cabinet. As mentioned earlier, the identification of bacteria was achieved using MALDI-TOF MS and 16S rRNA gene sequencing.

### Statistical analysis and reproducibility

Statistical analyses were performed using OECloud tools (<https://cloud.oebiotech.com/>) and open-source R software. α-diversity and microbial community comparisons were evaluated using ANOVA permutation and Kruskal-Wallis tests. Binary Jaccard distances were statistically analyzed using permutational multivariate analysis of variance (PERMANOVA) to assess differences in β-diversity. All statistical tests were two-sided, and results with *p* < 0.05 were considered statistically significant.

## References

1. García-García, María J. 2020. “A history of mouse genetics from fancy mice to mutations in every gene.” *Advances in Experimental Medicine and Biology* 1236: 1−38. https://doi.org/10.1007/978-981-15-2389-2_1

2. Tam, Wing Yip, Kwok-Kuen Cheung. 2020. “Phenotypic characteristics of commonly used inbred mouse strains.” *Journal of Molecular Medicine* 98: 1215−1234. https://doi.org/10.1007/s00109-020-01953-4

3. Sun, Xiaowei, Hua Zhang, Xiao Zhang, Wenmin Gao, Caiyun Zhou, Xuanxuan Kou, Jingxin Deng, Jiangang Zhang. 2024. “The cellular microbiome of visceral organs: an inherent inhabitant of parenchymal cells.” *Microorganisms* 12: 1333. https://doi.org/10.3390/microorganisms12071333

4. Greathouse, K. Leigh, James R. White, Ashely J. Vargas, Valery V. Bliskovsky, Jessica A. Beck, Natalia von Muhlinen, Eric C. Polley, et al. 2018. “Interaction between the microbiome and TP53 in human lung cancer.” *Genome Biology* 19: 123. https://doi.org/10.1186/s13059-018-1501-6

5. Pushalkar, Smruti, Mautin Hundeyin, Donnele Daley, Constantinos P. Zambirinis, Emma Kurz, Ankita Mishra, Navyatha Mohan, et al. 2018. “The pancreatic cancer microbiome promotes oncogenesis by induction of innate and adaptive immune suppression.” *Cancer Discovery* 8: 403−416. https://doi.org/10.1158/2159-8290.Cd-17-1134

6. Xue, Chen, Junjun Jia, Xinyu Gu, Lin Zhou, Juan Lu, Qiuxian Zheng, Yuanshuai Su, Shusen Zheng, Lanjuan Li. 2022. “Intratumoral bacteria interact with metabolites and genetic alterations in hepatocellular carcinoma.” *Signal Transduction and Targeted Therapy* 7: 335. https://doi.org/10.1038/s41392-022-01159-9

7. Urbaniak, Camilla, Gregory B. Gloor, Muriel Brackstone, Leslie Scott, Mark Tangney, Gregor Reid, H. Goodrich-Blair. 2016. “The microbiota of breast tissue and its association with breast cancer.” *Applied and Environmental Microbiology* 82: 5039−5048. https://doi.org/10.1128/aem.01235-16

8. Nejman, Deborah, Ilana Livyatan, Garold Fuks, Nancy Gavert, Yaara Zwang, Leore T Geller, Aviva Rotter-Maskowitz, et al. 2020. “The human tumor microbiome is composed of tumor type-specific intracellular bacteria.” *Science* 368: 973-80. https://doi.org/10.1126/science.aay9189.

9. Apostolou, Panagiotis, Aggeliki Tsantsaridou, Ioannis Papasotiriou, Maria Toloudi, Marina Chatziioannou, Gregory Giamouzis. 2011. “Bacterial and fungal microflora in surgically removed lung cancer samples.” *Journal of Cardiothoracic Surgery* 6: 137. https://doi.org/10.1186/1749-8090-6-137

10. Segal, Leopoldo N., Alexander V. Alekseyenko, Jose C. Clemente, Rohan Kulkarni, Benjamin Wu, Hao Chen, Kenneth I. Berger, et al. 2013. “Enrichment of lung microbiome with supraglottic taxa is associated with increased pulmonary inflammation.” *Microbiome* 1: 19. https://doi.org/10.1186/2049-2618-1-19

11. Ghebre, Michael A., Pee Hwee Pang, Sarah Diver, Dhananjay Desai, Mona Bafadhel, Koirobi Haldar, Tatiana Kebadze, et al. 2018. “Biological exacerbation clusters demonstrate asthma and chronic obstructive pulmonary disease overlap with distinct mediator and microbiome profiles.” *Journal of Allergy and Clinical Immunology* 141: 2027−2036.e2012. https://doi.org/10.1016/j.jaci.2018.04.013

12. Haldar, Koirobi, Leena George, Zhang Wang, Vijay Mistry, Mohammadali Yavari Ramsheh, Robert C. Free, Catherine John, et al. 2020. “The sputum microbiome is distinct between COPD and health, independent of smoking history.” *Respiratory Research* 21: 183. https://doi.org/10.1186/s12931-020-01448-3

13. Li, Ruomeng, Jing Li, Xikun Zhou. 2024. “Lung microbiome: new insights into the pathogenesis of respiratory diseases.” *Signal Transduction and Targeted Therapy* 9: 19. https://doi.org/10.1038/s41392-023-01722-y

14. Xie, Yifan, Feng Xie, Xiaoxue Zhou, Lei Zhang, Bing Yang, Jun Huang, Fangwei Wang, et al. 2022. “Microbiota in tumors: from understanding to application.” *Advanced Science* 9: e2200470. https://doi.org/10.1002/advs.202200470

15. Bertocchi, Alice, Sara Carloni, Paola Simona Ravenda, Giovanni Bertalot, Ilaria Spadoni, Antonino Lo Cascio, Sara Gandini, et al. 2021. “Gut vascular barrier impairment leads to intestinal bacteria dissemination and colorectal cancer metastasis to liver.” *Cancer Cell* 39: 708−724.e711. https://doi.org/10.1016/j.ccell.2021.03.004

16. Mekada, Kazuyuki, Atsushi Yoshiki. 2021. “Substrains matter in phenotyping of C57BL/6 mice.” *Experimental Animals* 70: 145−160. https://doi.org/10.1538/expanim.20-0158

17. Mekada, Kazuyuki, Kuniya Abe, Ayumi Murakami, Satoe Nakamura, Hatsumi Nakata, Kazuo Moriwaki, Yuichi Obata, Atsushi Yoshiki. 2009. “Genetic differences among C57BL/6 substrains.” *Experimental Animals* 58: 141−149. https://doi.org/10.1538/expanim.58.141

18. Dumont, Beth L., Anne Yoder. 2019. “Significant strain variation in the mutation spectra of inbred laboratory mice.” *Molecular Biology and Evolution* 36: 865−874. https://doi.org/10.1093/molbev/msz026

19. Åhlgren, Johanna, Vootele Voikar. 2019. “Experiments done in Black-6 mice: what does it mean?” *Lab Animal* 48: 171−180. https://doi.org/10.1038/s41684-019-0288-8

20. Uchimura, Arikuni, Mayumi Higuchi, Yohei Minakuchi, Mizuki Ohno, Atsushi Toyoda, Asao Fujiyama, Ikuo Miura, Shigeharu Wakana, Jo Nishino, Takeshi Yagi. 2015. “Germline mutation rates and the long-term phenotypic effects of mutation accumulation in wild-type laboratory mice and mutator mice.” *Genome Research* 25: 1125−1134. https://doi.org/10.1101/gr.186148.114

21. Lagier, J. C., F. Armougom, M. Million, P. Hugon, I. Pagnier, C. Robert, F. Bittar, et al. 2012. “Microbial culturomics: paradigm shift in the human gut microbiome study.” *Clinical Microbiology and Infection* 18: 1185−1193. https://doi.org/10.1111/1469-0691.12023

22. Tidjani Alou, M., Sabrina Naud, Saber Khelaifia, Marion Bonnet, Jean-Christophe Lagier, Didier Raoult. 2021. “State of the art in the culture of the human microbiota new interests and strategies.” *Clinical Microbiology Reviews* 34: e00129-19. https://doi.org/10.1128/CMR.00129-19

23. Wang, Shi, Eli Meyer, John K. McKay, Mikhail V. Matz. 2012. “2b-RAD: a simple and flexible method for genome-wide genotyping.” *Nature Methods* 9: 808−810. https://doi.org/10.1038/nmeth.2023

24. Sun, Zheng, Shi Huang, Pengfei Zhu, Lam Tzehau, Helen Zhao, Jia Lv, Rongchao Zhang, et al. 2022. “Species-resolved sequencing of low-biomass or degraded microbiomes using 2bRAD-M.” *Genome Biology* 23: 36. https://doi.org/10.1186/s13059-021-02576-9

25. Lagier, Jean-Christophe, Saber Khelaifia, Maryam Tidjani Alou, Sokhna Ndongo, Niokhor Dione, Perrine Hugon, Aurelia Caputo, et al. 2016. “Culture of previously uncultured members of the human gut microbiota by culturomics.” *Nature Microbiology* 1: 16203. https://doi.org/10.1038/nmicrobiol.2016.203


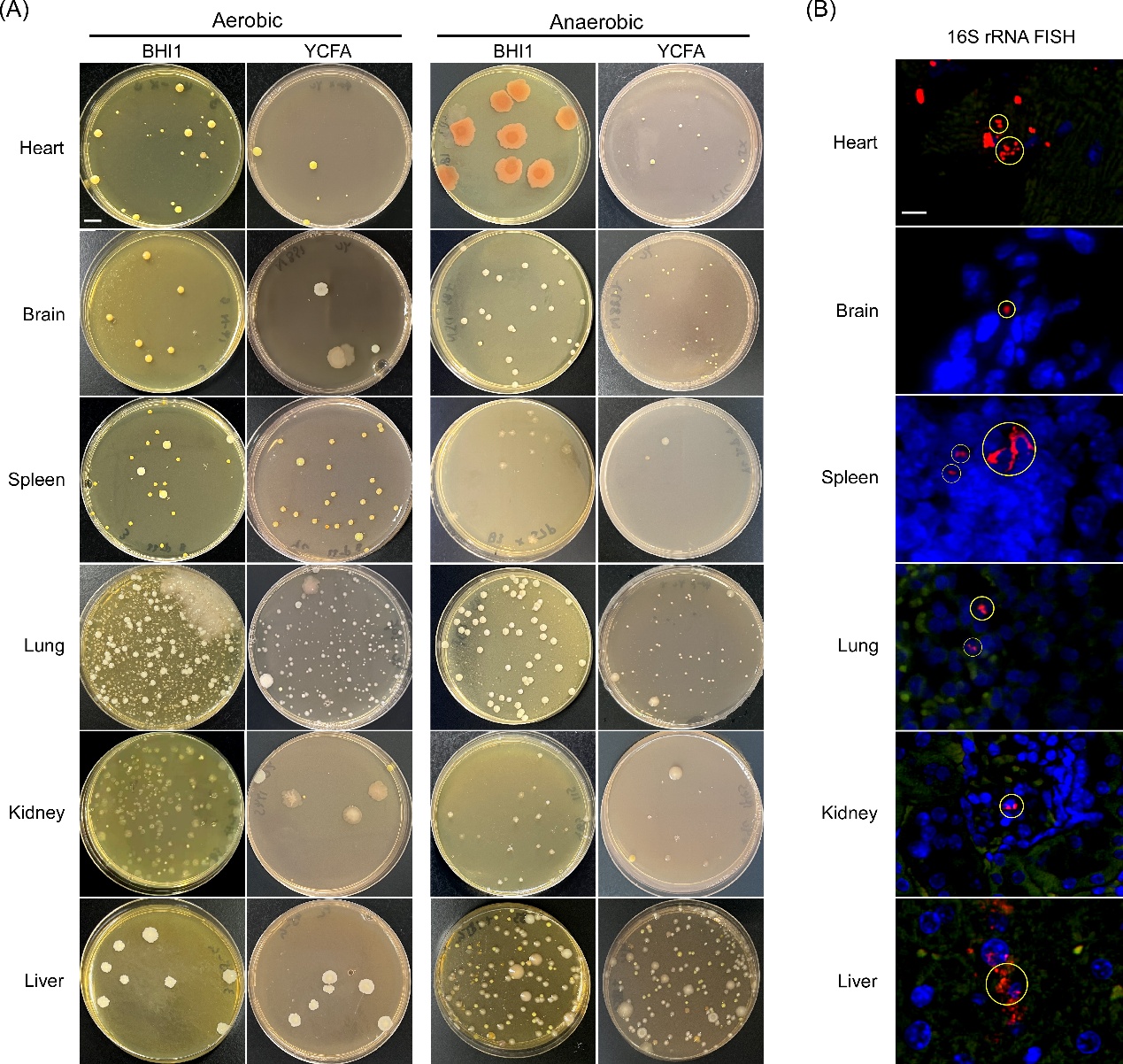


**Figure S1** **Representative examples of culture plates and FISH images for microbial detection in mouse brain, heart, kidney, liver, lung, and spleen tissues.** (A) Microbial colonies on BHI1 and YCFA medium plates. After the mice were humanely euthanized, their organs (brain, heart, kidney, liver, lung, and spleen) were immediately used for culturomics analysis. The plates were cultured under aerobic or anaerobic condition at 37^o^C for 5 days. BHI1, Brain Heart Infusion Agar + L-cysteine hydrochloride hydrate + hemin + vitamin K. YCFA, Yeast extract-Casein hydrolysate Fatty Acids. Scale bar: 1 cm. (B) Representative images for fluorescence in situ 16S rRNA-hybridization (FISH) assays using the bacterial 16S rRNA probe in mouse tissues. The dissected tissues were fixed with 10% formalin, embedded in paraffin, and sectioned. Tissue sections were hybridized with Cy3-labeled probe EUB338 (5'-GCTGCCTCCCGTAGGAGT-3') (red) and 6-FAM labeled nonspecific complement probe (5'-CGACGGAGGGCATCCTCA-3') (green). The latter was used as a negative control to rule out non-specific hybridization. After hybridization, the sections were washed to remove the hybridization solution, and nuclei were counterstained with diamidino-phenyl-indole (DAPI). The images were captured using fluorescent microscopy. FAM (488) glows green by excitation wavelength 465-495 nm and emission wavelength 515-555 nm; CY3 glows red by excitation wavelength 510-560 nm and emission wavelength 590 nm. Circles indicate bacteria stained by the FISH probe. Scale bar: 10 μm. Representative images for each tissue are shown.

**
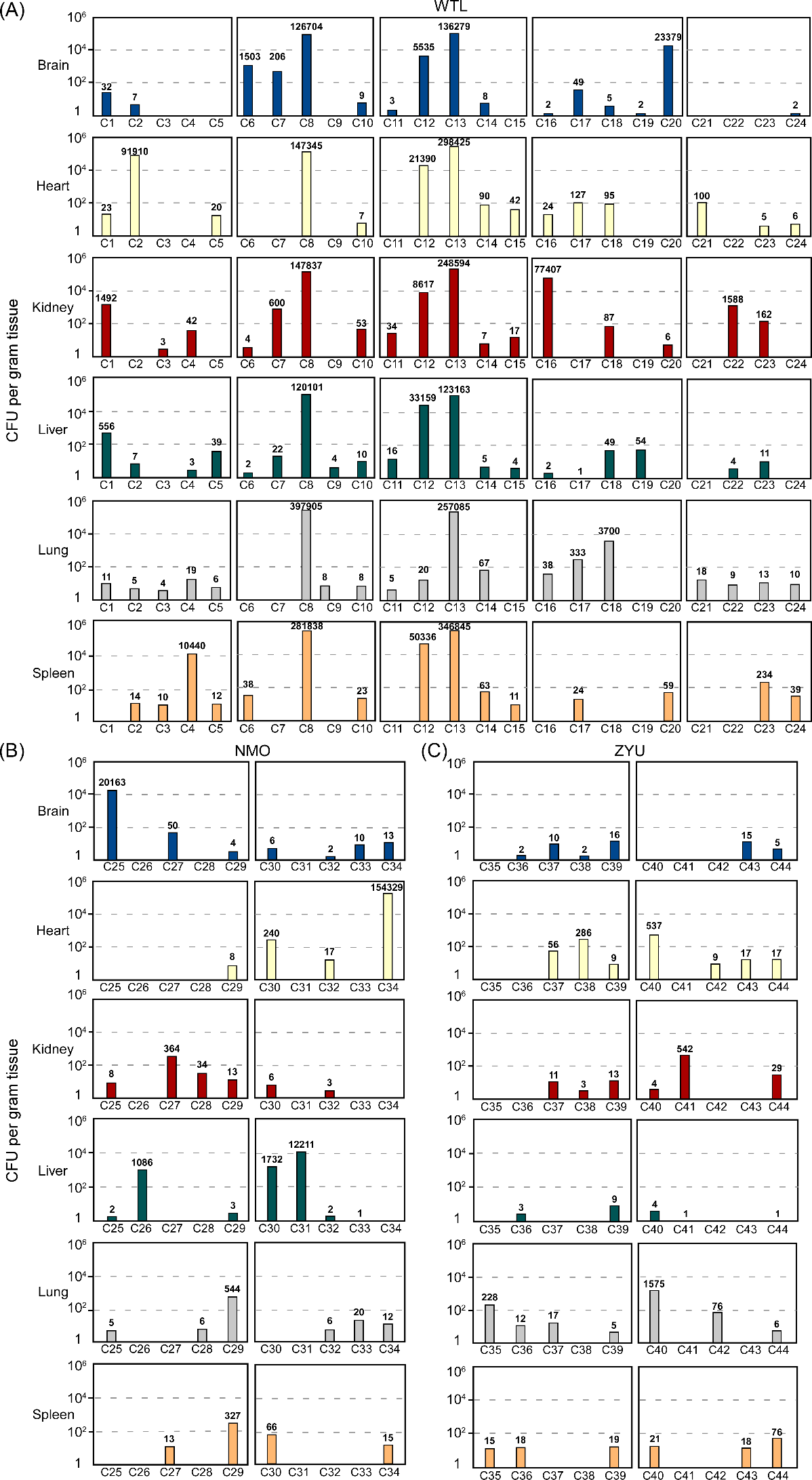
**

**Figure S2** **Abundance of microbes detected in the brain, heart, kidney, liver, lung, and spleen tissues of C57BL/6J mice** **based on culturomics assays.** Microbial burdens were evaluated in a total of 44 C57BL/6J mice purchased from three experimental animal providers in China: WTL (A), NMO (B), and ZYU (C). Upon arrival, the mice were immediately euthanized, and their organs were used for microbial burden analysis. After complete anesthesia, the body surface was sterilized three times with 75% ethanol. The microbial abundance (CFU/g tissue) in the six organs was evaluated at 37^°^C using seven optimized cultural media. CFU, colony-forming unit. The column colors represent specific organs, with the numbers on the columns indicating CFU values. Detailed information about the mice used is provided in **Table S1**. C1-C44, mouse codes ranked by treatment order. C: C57BL/6J.


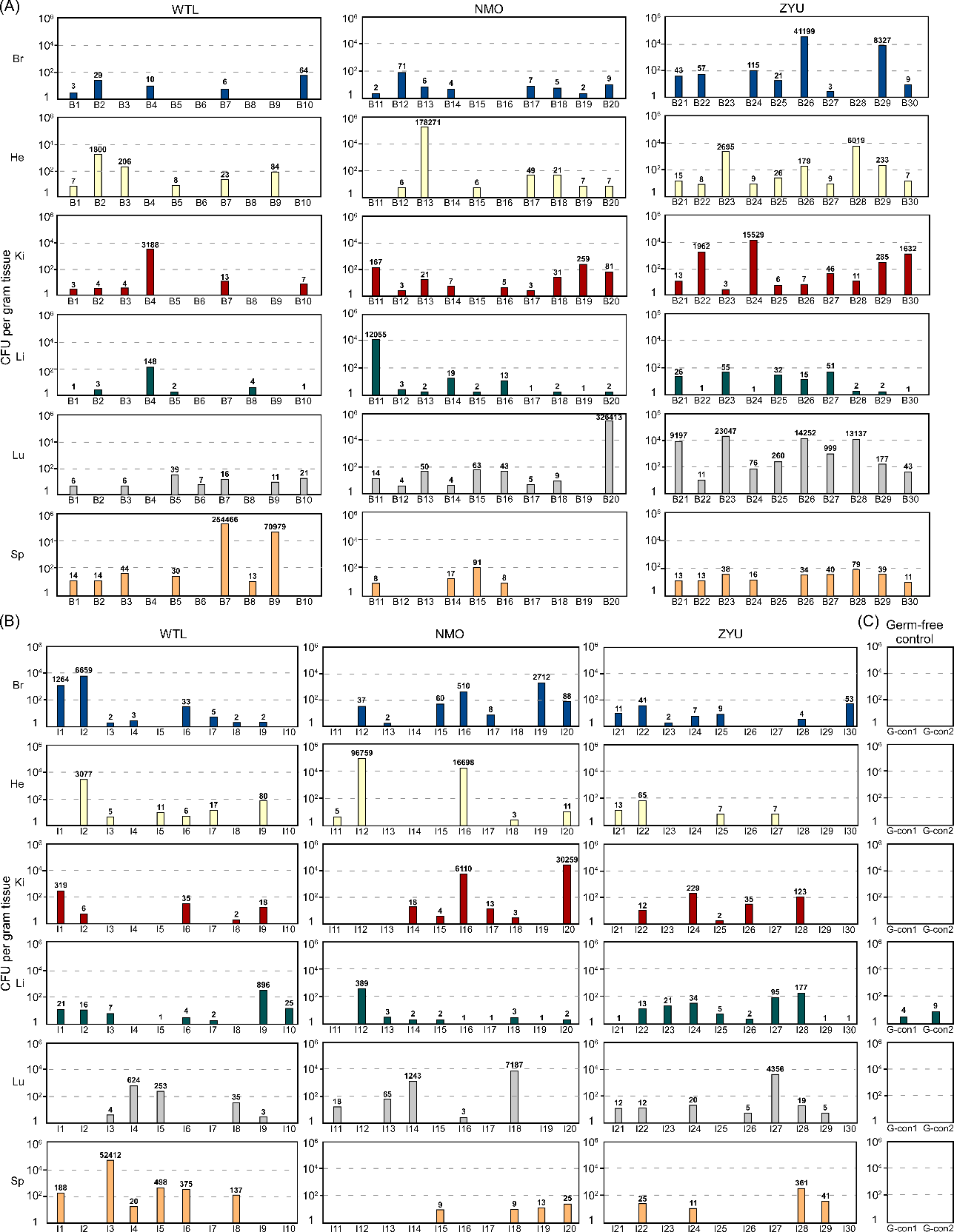


**Figure S3** **Abundance of microbes detected in the brain, heart, kidney, liver, lung, and spleen tissues of BALB/c (A), ICR (B) and germ-free (C) mice** **based on culturomics assays.** (A) and (B) A total of 60 mice (30 BALB/c and 30 ICR) were purchased from three experimental animal providers in China (WTL, NMO, and ZYU) and examined. The microbial abundance (CFU/g tissue) of the six organs of each mouse was evaluated at 37^°^C using the 7 optimal cultural media. (C) Two germ-free mice gavaged with sterile phosphate-buffered saline (PBS) were examined. The six organs used include brain (Br), heart (He), kidney (Ki), liver (Li), lung (Lu), spleen (Sp). The column color represents a specific organ, and the numbers on the columns indicate the CFU values. Detailed methods are described in **Figure S2**. The mouse details are shown in **Table S1**. Mice used: B1-B30, I1-I30, and G-con1, G-con2; mouse codes ranked by treatment order. B: BALB/c; I: ICR; G-con, Germ-free control (C57BL/6J).


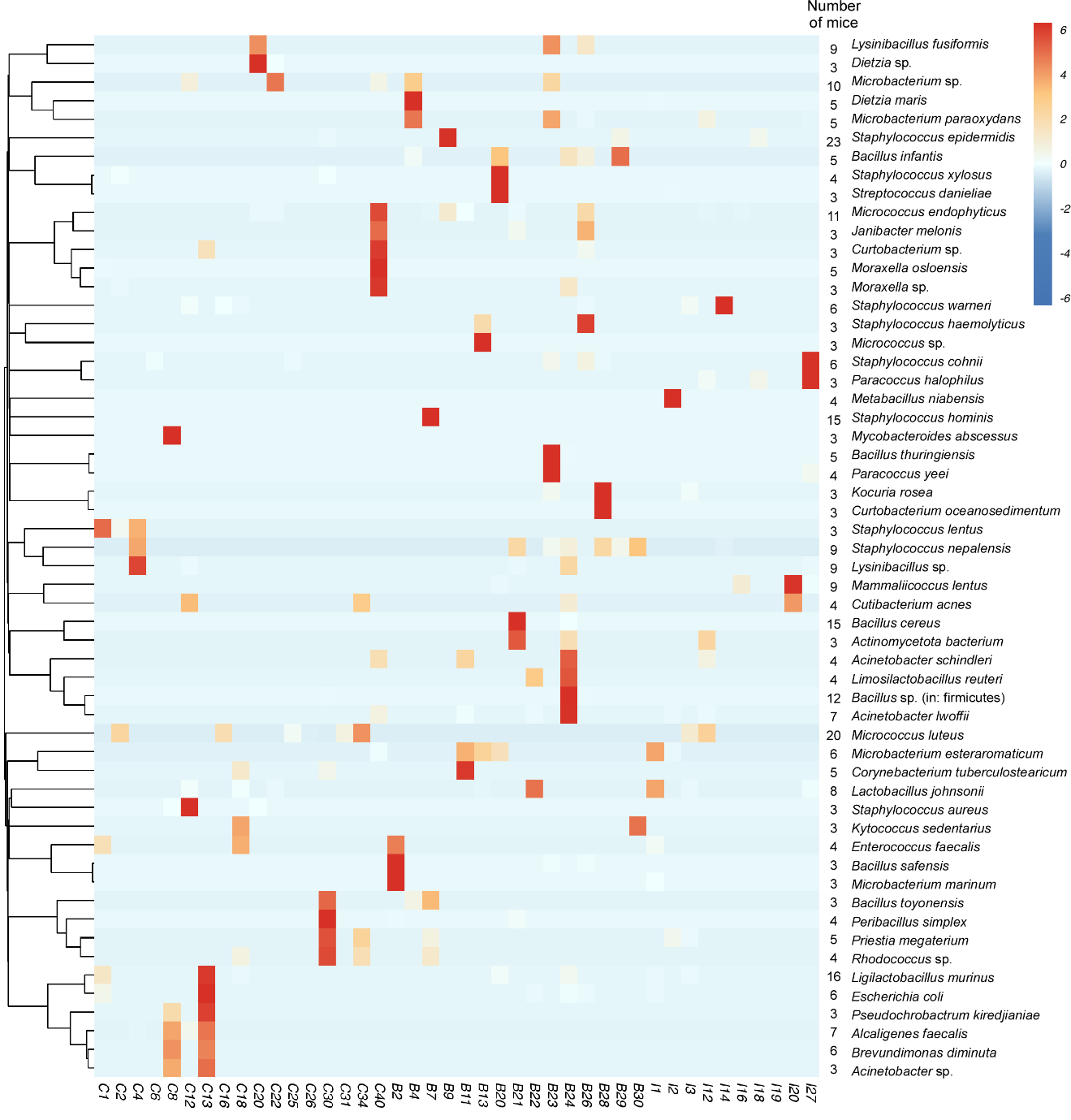


**Figure S4** **Composition and abundance of microbial species isolated by culturomics assays from six organ tissues of mice with a high microbial burden.** 42 mice with a microbial burden higher than 1 × 10^3^ CFU/g tissue in at least one organ were detected. The heatmap depicts the composition and abundance of microbial species detected in the organs of at least three mice. Microbial species and number of mice harboring the corresponding species were displayed on right side of the heatmap. For species identification, two representative colonies of each distinct colony morphology on each plate were selected. The CFU of each microbial species in an organ was estimated based on similar colony phenotype on the same plate. The heatmap, representing relative abundance, was calculated as the average CFU/g tissue across the six organs. The heatmap representing relative abundance, was generated using OECloud tools (<https://cloud.oebiotech.com>). The intensity of red coloration indicates the relative microbial abundance. The dendrogram illustrates the phylogenetic relationships of the microbial cohorts. Mouse codes shown at the bottom of the image correspond to the treatment order. C, C57BL/6J; B, BALB/c; and I, ICR. This figure is associated with **Figures S2, S3** and **Tables S3, S4** and **S5**.

**
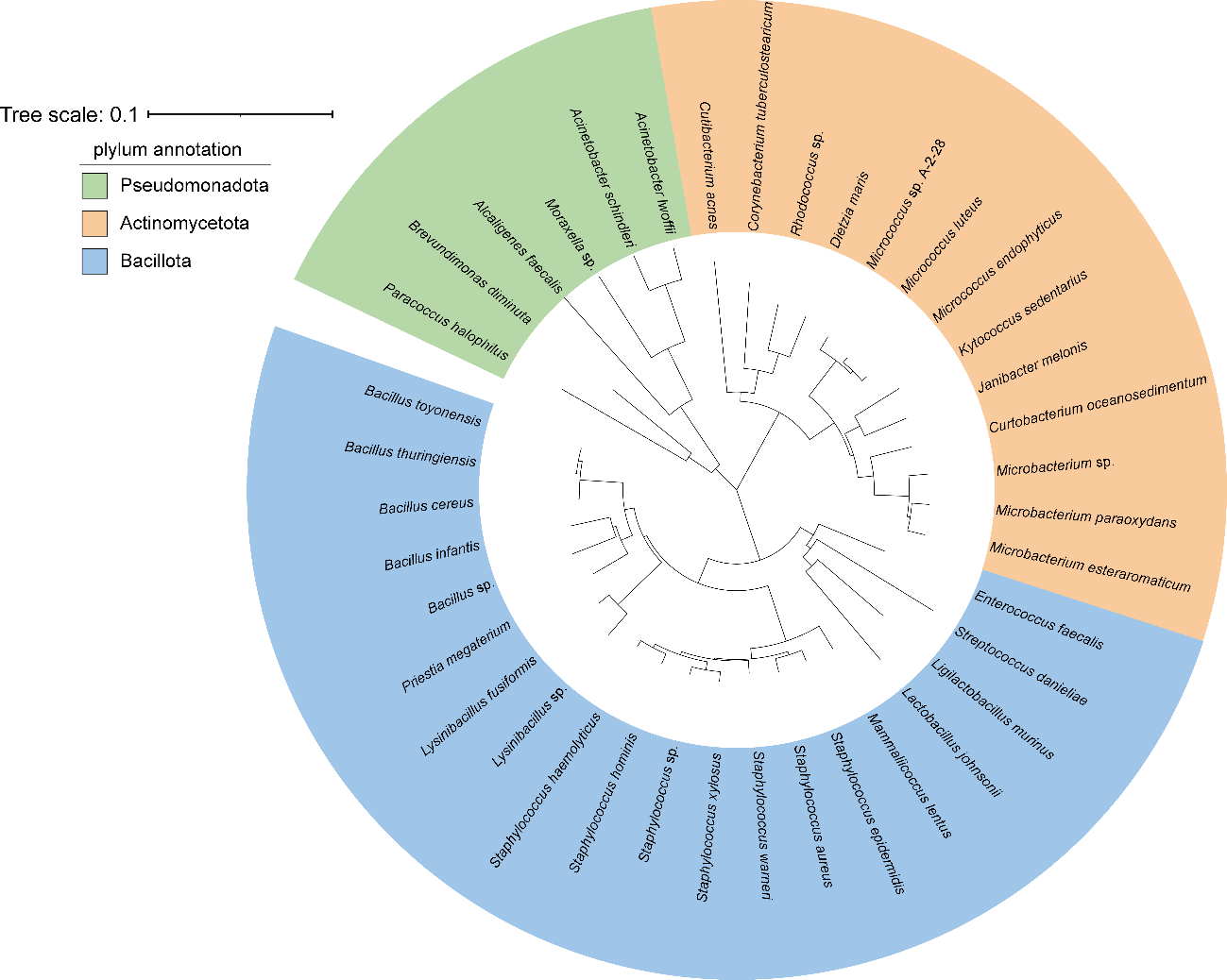
**

**Figure S5** **Phylogenetic tree of the representative species enriched in the mouse brain, heart, kidney, liver, lung, and spleen tissues.** The species of Pseudomonadota*,* Actinomycetota, and Bacillota are highlighted in green, orange, and blue, respectively. The absolute total branch length is indicated at the bottom. The phylogenetic tree was based on data from MEGA11 and visualized using the iTOL platform (<https://itol.embl.de/>). 16S rDNA sequences of the representative species are shown in **Table S4**.


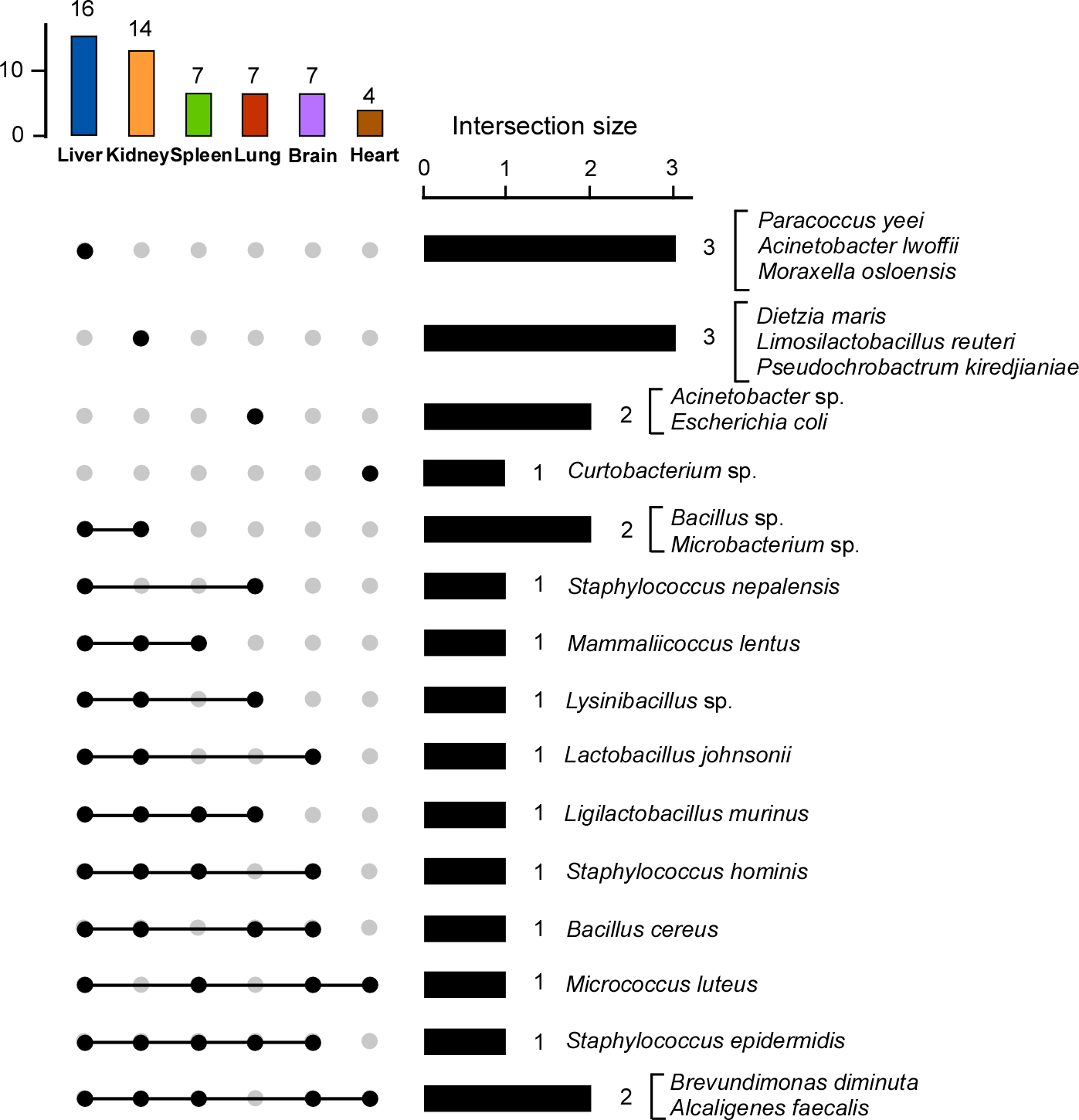


**Figure S6** **Unique and overlap species among different organs of 42 mice with a microbial burden higher than 10^3^ CFU/g tissue.** UpSet plots show the number of common microbial species among the six organs. Rows represent different combinations of organs. The shared or specific species were displayed on right side of the plot. The detailed information for the shared species among the organs is presented in **Table S5**.


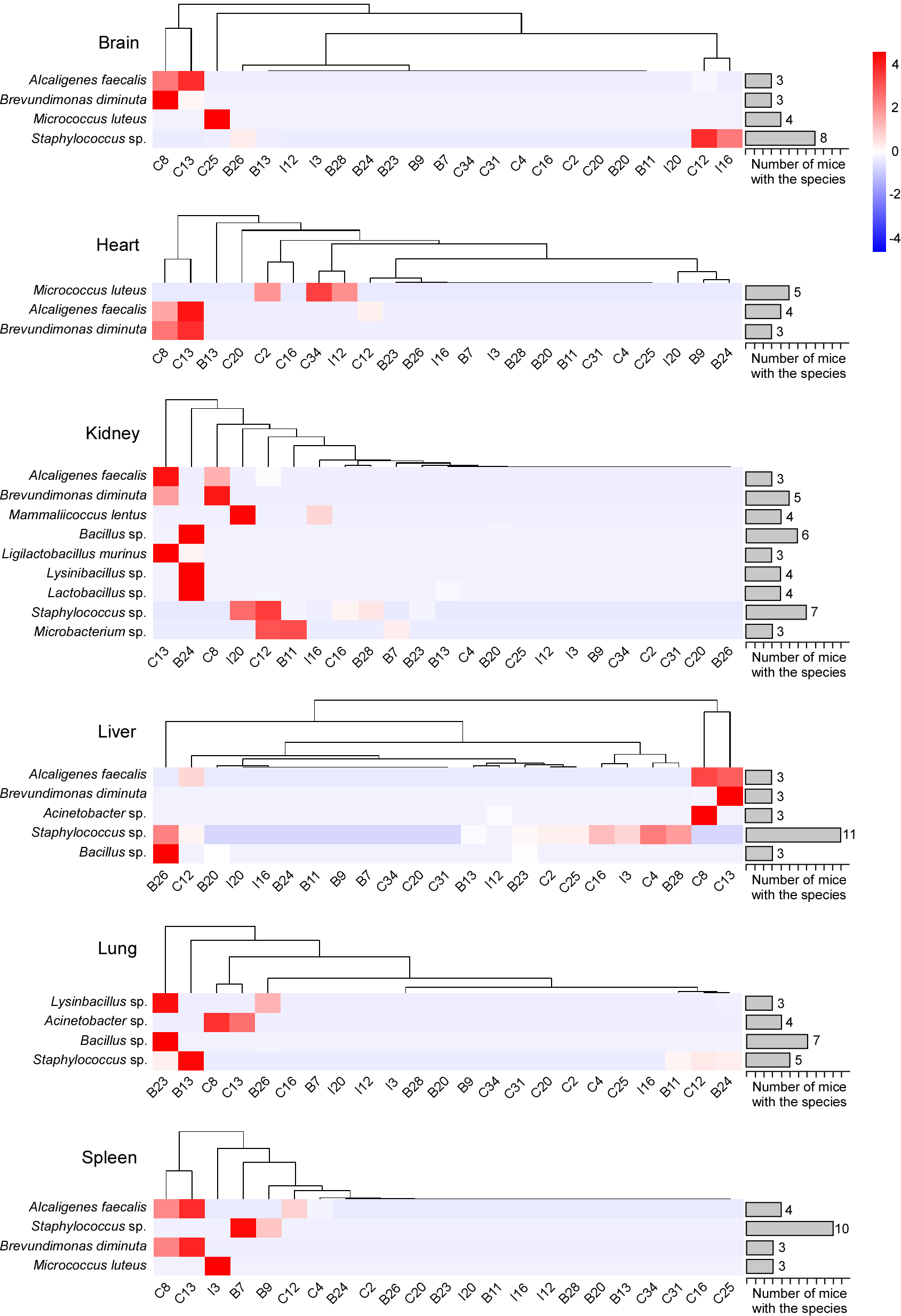


**Figure S7** **Most frequently isolated microbial species from different organs of the 23 mice with a high microbial burden (> 10^4^ CFU/g tissue).** The mice analyzed in this figure include C2, C4, C8, C12, C13, C16, C20, C25, C31, C34, B7, B9, B11, B13, B20, B23, B24, B26, B28, I3, I12, I16, and I20. The heatmap shows the composition and abundance of microbial species isolated from the brain, heart, kidney, liver, lung, and spleen tissues of at least three mice with a high microbial burden. Microbial abundance (CFU/g tissue) was evaluated for each organ of the individual mice. The intensity of red coloration indicates the relative microbial abundance. The dendrogram shows the phylogenetic relationships of the microbial cohorts. Number of mice harboring the corresponding species was displayed on right side of the plots. The relative microbial abundance was calculated based on the CFU of each microbial species in the corresponding organ. The heatmap was generated using OECloud tools at <https://cloud.oebiotech.com>. This figure is associated with **Figures S2, S3** and **S4**. The detailed information for the mice and microbial species, as well as CFU, is provided in **Tables S3** and **S5**.


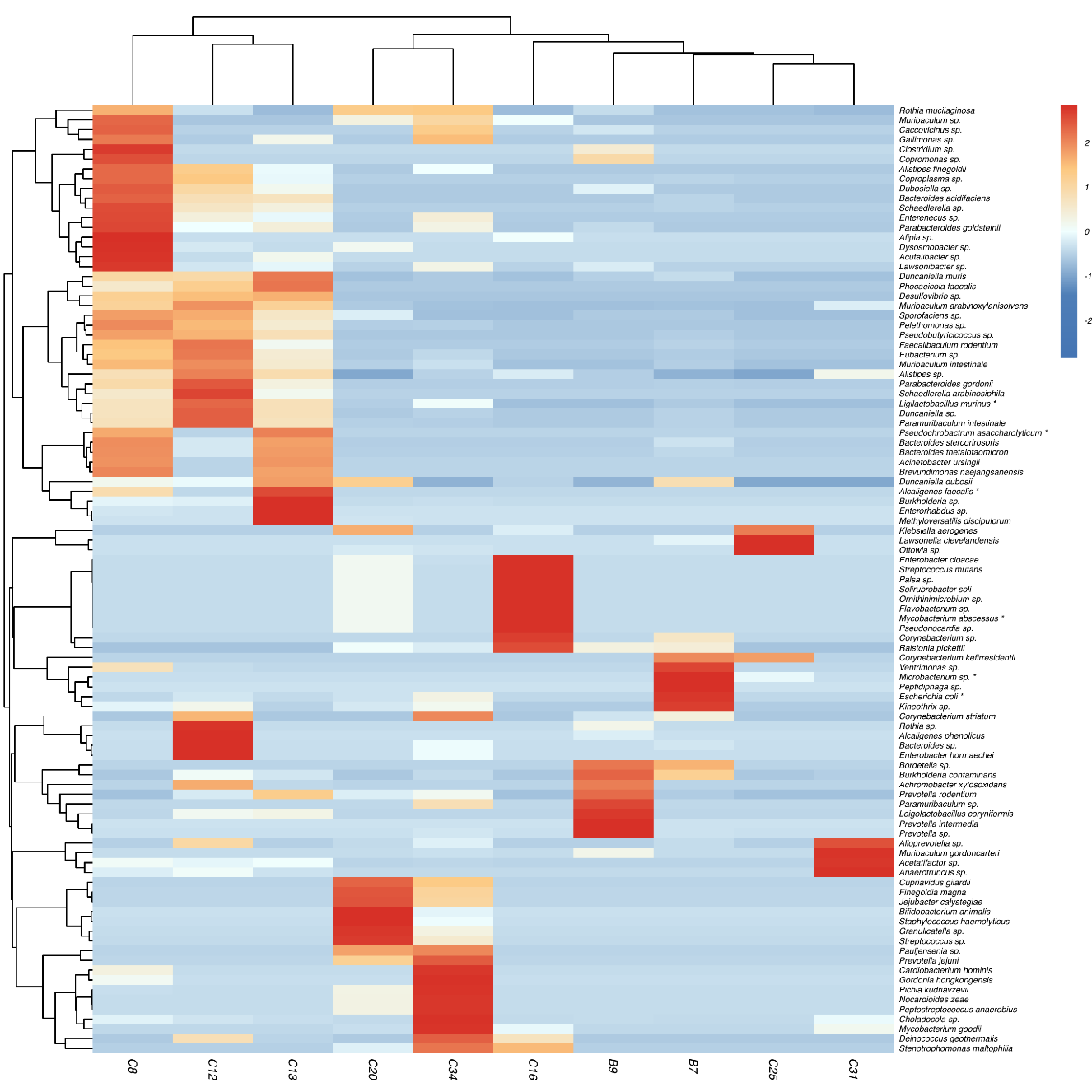


**Figure S8** **Composition and abundance of microbial species in the 10 mice** **with high microbial abundance detected by metagenomics assays.** This analysis includes mice C8, C12, C13, C16, C20, C25, C31, C34, B7, and B9. The heatmap shows the composition and abundance of microbial species with a relative abundance > 10^-4^ detected in at least two mice by metagenomics assays. The relative abundance of microbial species across brain, heart, kidney, liver, lung, and spleen tissues of each mouse was based on the average read coverage of 2bRAD markers. All sample data were filtered to remove mock-derived (no tissue) data. The intensity of red coloration indicates the relative microbial abundance, and the dendrogram shows the phylogenetic relationships of the microbial cohorts. The * symbol marks species identified by both culturomics and metagenomics assays. Detailed information for the mice, microbial species, and relative abundance is provided in **Table S6**.


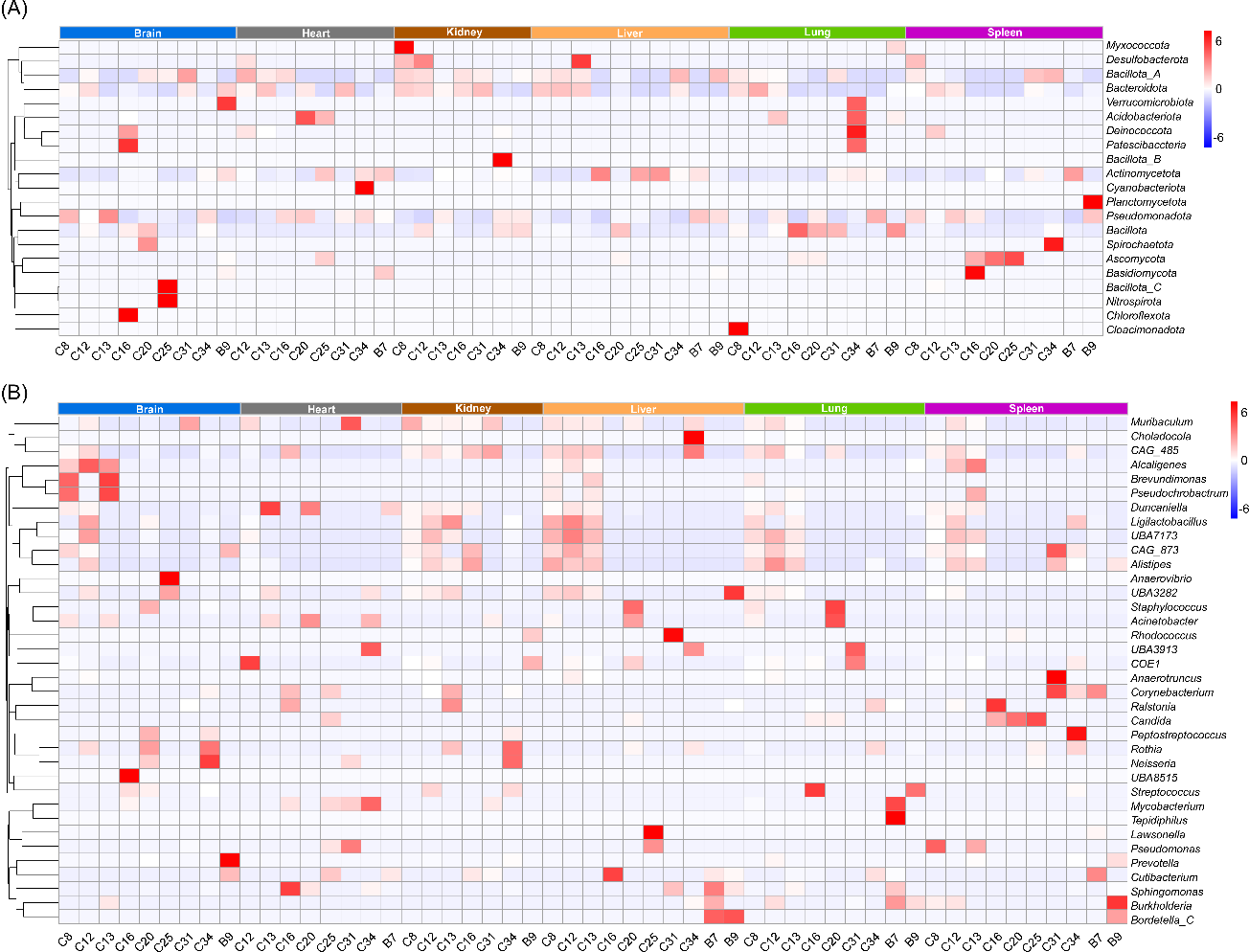


**Figure S9** **Taxonomic composition of microbes in each organ of the 10 high-burden mice detected by metagenomics assays.** Mice analyzed in this figure include C8, C12, C13, C16, C20, C25, C31, C34, B7, and B9. The relative abundance of microbial species was calculated as described in **Figure S8**. All sample data were filtered to remove mock-derived (no tissue) data. The intensity of red coloration indicates the relative microbial abundance, with darker red representing higher abundance. The dendrogram shows the phylogenetic relationships of the microbial cohorts. Organ information is indicated at the top of the images. (A) Taxonomic composition at the phylum level. (B) Taxonomic composition at the genus level. Detailed information for the mouse strains, microbial species, and relative abundance is provided in **Table S7**.


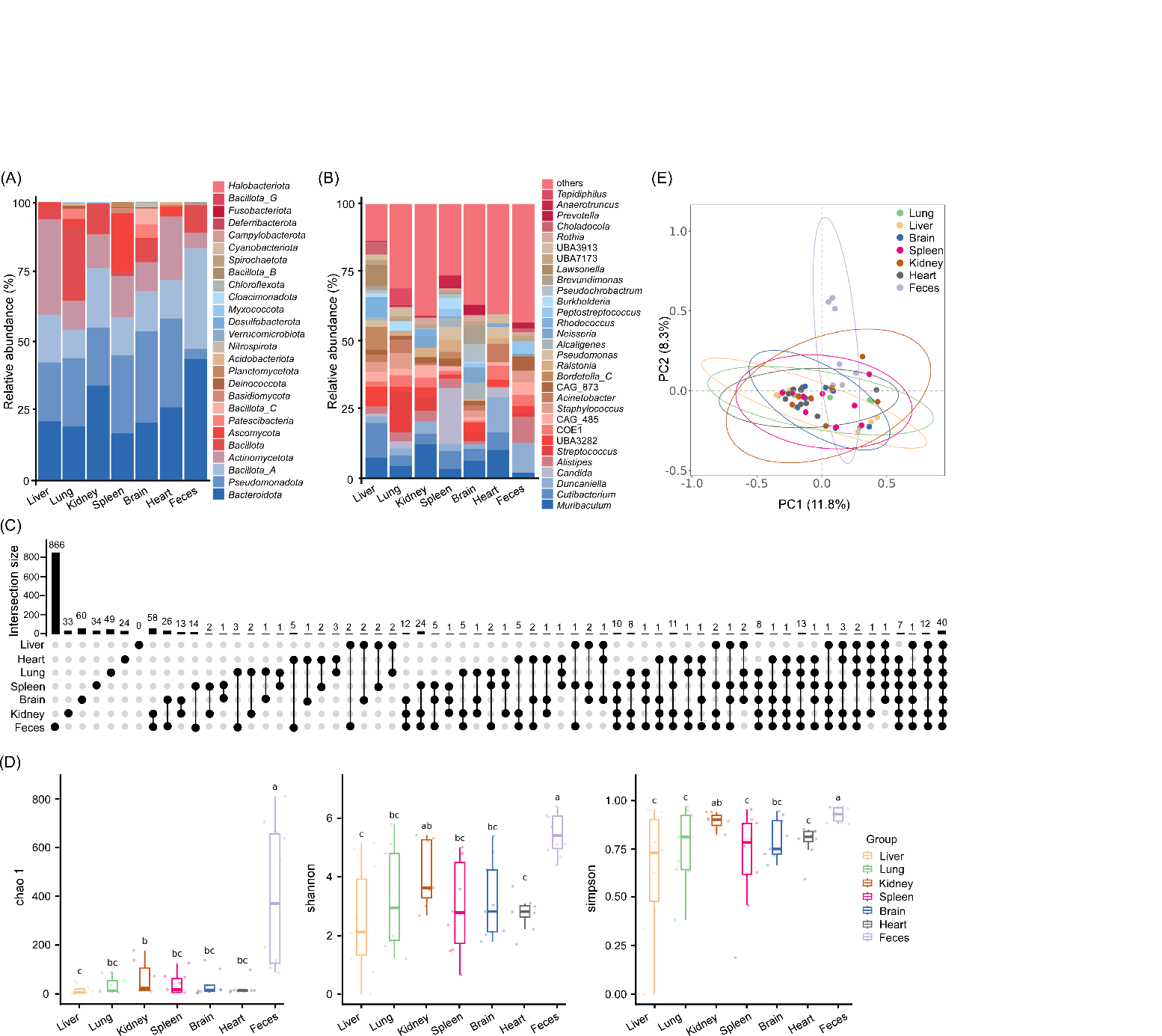


**Figure S10** **Comparative analysis of the microbiomes between the organs and feces detected in 10 high-burden mice.** (A, B) Comparative analysis of the dominant species detected in the organs and feces at the phylum (A) and genus (B) levels. (C) UpSet plots showing the number of common microbial species between the organ and fecal microbiomes. (D) The organ and fecal microbiomes in healthy mice were analyzed for α-diversity measures (Chao1, Shannon, and Simpson). ***p* < 0.01. (E) Weighted PCoA plots based on Binary_Jaccard dissimilarity matrix (95% CI ellipses). Mice detected included C8, C12, C13, C16, C20, C25, C31, C34, B7, and B9.


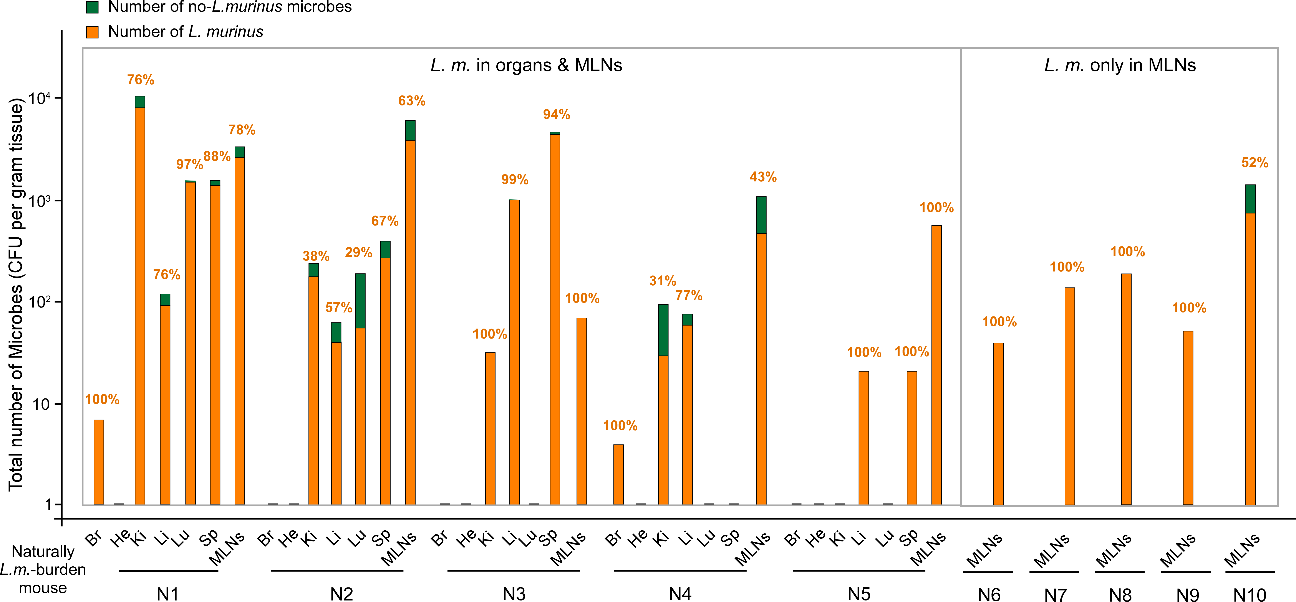


**Figure S11** **The distribution of** ***L. murinus* in the mouse organs and MLNs.** Microbial burdens were evaluated in a total of 10 C57BL/6J mice purchased from ZYU. Upon arrival, the mice were immediately euthanized, and their organs were used for microbial burden analysis. After complete anesthesia, the body surface was sterilized three times with 75% ethanol. The microbial abundance (CFU/g tissue) in the six organs and MLNs was evaluated at 37^°^C using MRS media (**Table S2**). CFU, colony-forming unit. Columns represent number of microbes (CFU/g tissue) detected in the organs. The orange columns represent the burdens of *L. murinus* (CFU/g tissue) and the green columns represent the burdens of other microbes (CFU/g tissue). The percentages on the columns indicate the proportions of *L. murinus* CFU. Six organs include brain (Br), heart (He), kidney (Ki), liver (Li), lung (Lu), spleen (Sp). MLNs, mesenteric lymph nodes. The left panel indicates mice that harbor *L. murinus* both in the organs and in MLNs. The right panel indicates mice that harbor *L. murinus* only in MLNs. Detailed information for the mice, microbial species, and relative abundance is provided in **Source Data file**.

**
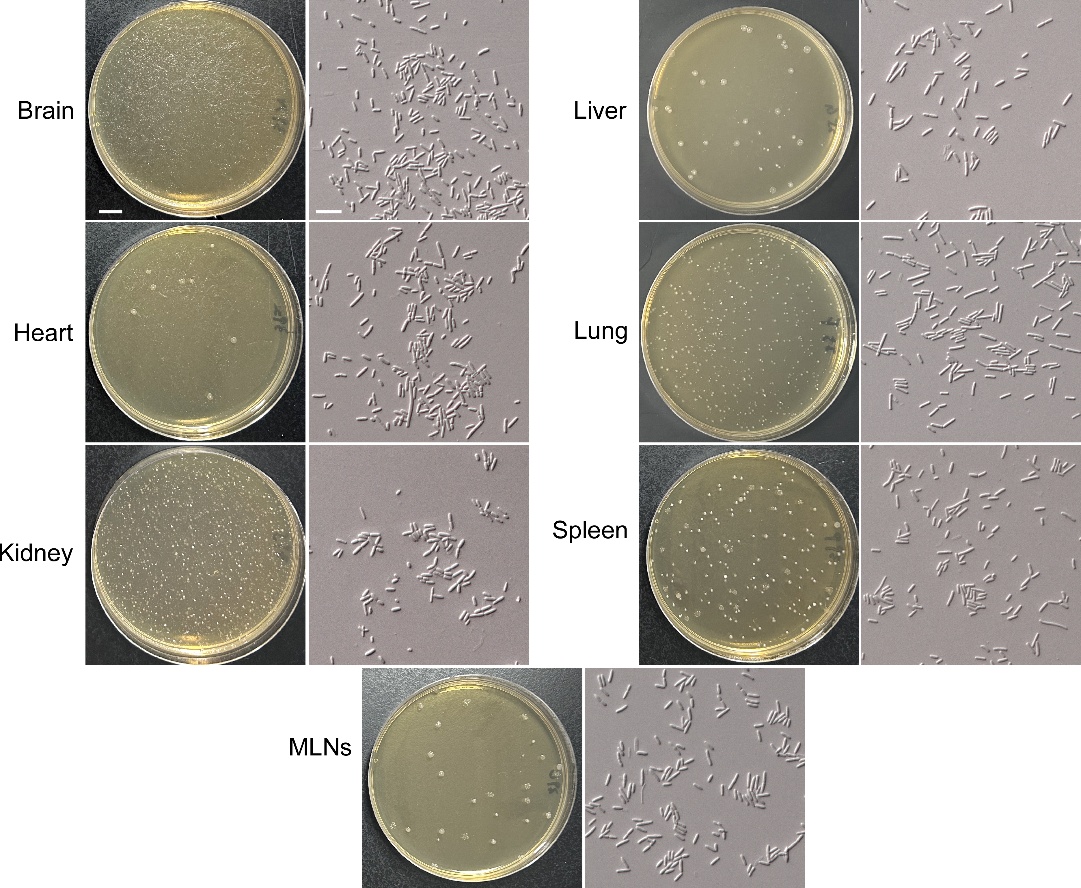
**

**Figure S12** **Representative examples of culture phenotype for *Ligilactobacillus murinus* detection in the organs of the** **naturally microbe-burden mice.**

Colonial and cellular images of *L*. *murinus* isolates derived from different organs of the naturally microbe-burden mice. MRS medium was used. 10 naturally microbe-burden mice (C57BL/6J, 7 weeks, male) were disinfected, euthanized, and dissected. Six organs including brain, heart, kidney, liver, lung, and spleen, and MLNs of each mouse were harvested, weighed, and homogenized for microbial isolation. The plates were cultured under aerobic condition at 37^o^C for 5 days. Scale bar for colonies, 1 cm; Scale bar for cells, 20 μm.
